# Design of a Stable Cyclic Peptide Analgesic Derived from Sunflower Seeds that Targets the κ-Opioid Receptor for the Treatment of Chronic Abdominal Pain

**DOI:** 10.1021/acs.jmedchem.1c00158

**Published:** 2021-06-24

**Authors:** Edin Muratspahić, Nataša Tomašević, Johannes Koehbach, Leopold Duerrauer, Seid Hadžić, Joel Castro, Gudrun Schober, Spyridon Sideromenos, Richard J. Clark, Stuart M. Brierley, David J. Craik, Christian W. Gruber

**Affiliations:** † Center for Physiology and Pharmacology, Institute of Pharmacology, Medical University of Vienna, 1090 Vienna, Austria; ‡ Institute for Molecular Bioscience, Australian Research Council Centre of Excellence for Innovations in Peptide and Protein Science, The University of Queensland, Brisbane, Queensland 4072, Australia; § School of Biomedical Sciences, Faculty of Medicine, The University of Queensland, Brisbane, Queensland 4072, Australia; ∥ Visceral Pain Research Group, College of Medicine and Public Health, Flinders Health and Medical Research Institute (FHMRI), Flinders University, Bedford Park, South Australia 5042, Australia; ⊥ Hopwood Centre for Neurobiology, Lifelong Health Theme, South Australian Health and Medical Research Institute (SAHMRI), North Terrace, Adelaide, South Australia 5000, Australia; # Center for Physiology and Pharmacology, Department of Neurophysiology and Neuropharmacology, Medical University of Vienna, 1090 Vienna, Austria; ¶ Discipline of Medicine, University of Adelaide, North Terrace, Adelaide, South Australia 5000, Australia

## Abstract

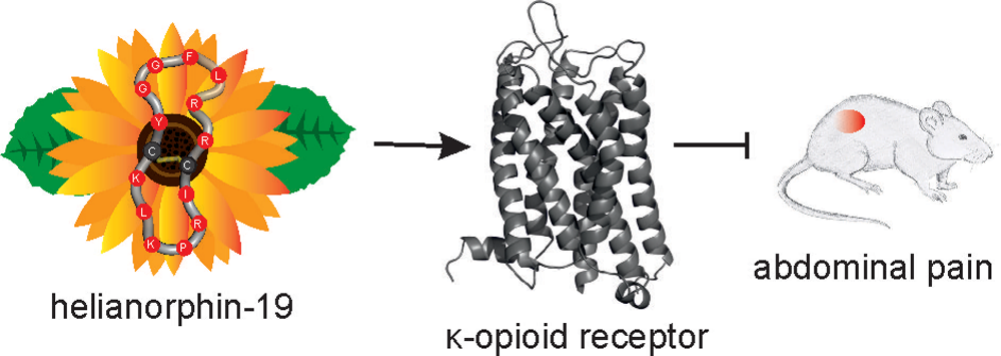

The rising opioid crisis has become a worldwide societal and public health burden, resulting from the abuse of prescription opioids. Targeting the κ-opioid receptor (KOR) in the periphery has emerged as a powerful approach to develop novel pain medications without central side effects. Inspired by the traditional use of sunflower (*Helianthus annuus*) preparations for analgesic purposes, we developed novel stabilized KOR ligands (termed as helianorphins) by incorporating different dynorphin A sequence fragments into a cyclic sunflower peptide scaffold. As a result, helianorphin-19 selectively bound to and fully activated the KOR with nanomolar potency. Importantly, helianorphin-19 exhibited strong KOR-specific peripheral analgesic activity in a mouse model of chronic visceral pain, without inducing unwanted central effects on motor coordination/sedation. Our study provides a proof of principle that cyclic peptides from plants may be used as templates to develop potent and stable peptide analgesics applicable via enteric administration by targeting the peripheral KOR for the treatment of chronic abdominal pain.

## Introduction

**Figure 1:**
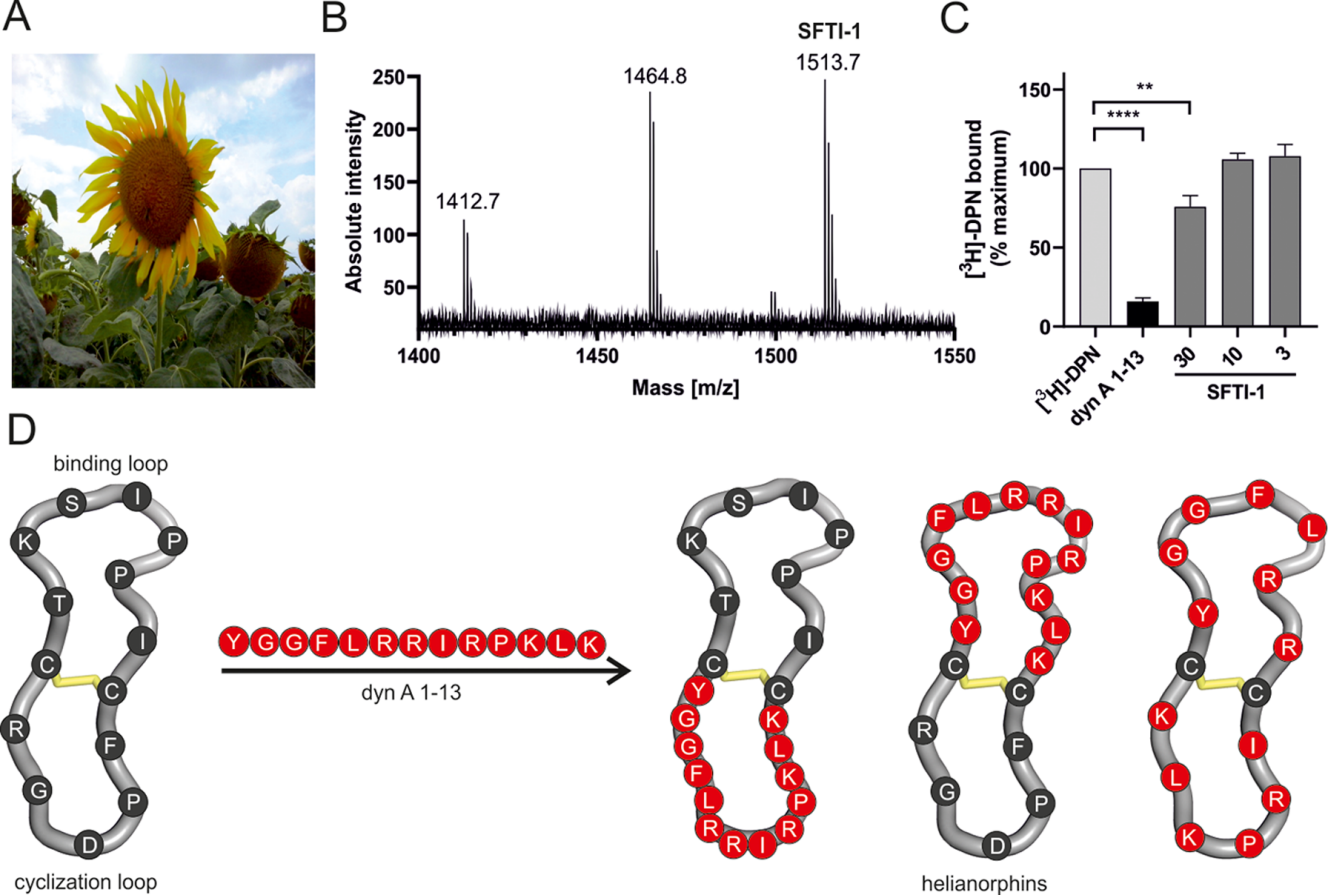
(A) Image of a representative sunflower (*Helianthus annuus*) [photograph with permission of Thorsten Medwedeff]. (B) MALDI-TOF mass spectrometry of a sunflower seed extract yielded a spectrum containing the monoisotopic mass (1513.7 *m*/*z*) of sunflower trypsin inhibitor-1 (SFTI-1). (C) Three concentrations of SFTI-1 (in μM) were tested by radioligand displacement (1 nM [^3^*H*]-diprenorphine, DPN) using HEK293 cell membranes stably expressing the mouse KOR. Specific binding was defined by subtracting nonspecific binding from total binding. Data are shown as mean ± SD (*n* = 3) and are normalized to the percentage of maximum binding. Dynorphin A (dyn A) 1–13 was used as a positive control (10 nM, *n* = 3). Statistical difference was analyzed using an unpaired *t*-test (*P < 0.05; ***P < 0.001). (D) Schematic illustration of the SFTI-1 (PDB: 1jbl) grafting strategy with different dyn A 1–13 epitopes. Dyn A 1–13 was incorporated into either the cyclization loop or binding loop or was divided into two fragments and inserted into both loops. Dyn A 1–13 residues are shown in red, whereas SFTI-1 residues are colored in dark gray. Disulfide bonds are shown in yellow.

G protein-coupled receptors (GPCRs) continue to be among the most important drug targets, with small-molecule ligands of these receptors currently dominating the pharmaceutical market.^1^ The development of peptide therapeutics targeting GPCRs has been limited by poor pharmacokinetics (metabolic instability, short half-life, and rapid clearance) and lack of oral bioavailability (low gastrointestinal stability and membrane permeability).^2^ Strategies to tackle these challenges of peptides are thus urgently required. In recent years, nature-derived peptides have gained momentum in the GPCR drug discovery and development pipeline,^3^ including those for gastrointestinal disorders.^4^ Notably, plant-derived peptides offer the potential to design and develop potent GPCR ligands with improved pharmacological properties.^5^ Due to their exceptional stability and plasticity to accommodate sequence variations without affecting the overall three-dimensional structure, cyclic cystine-rich peptides such as cyclotides have been successfully utilized in molecular “grafting” applications. For instance, this approach has led to the design of potent, stable, and selective peptide ligands for the melanocortin 4 receptor,^6^ bradykinin B1 receptor,^7^ CXC-motif-chemokine receptor 4,^8^ and MAS 1 receptor.^9^ However, a drawback of cyclotide scaffolds is their sometimes low yield in oxidative folding, which may lead to high synthesis costs or even failure of the product.^10^ By contrast, the sunflower trypsin inhibitor-1 (SFTI-1) is a 14-residue cyclic peptide derived from the seeds of sunflowers (Figure 1A,B) that is stabilized by one disulfide bond, thus exhibiting a less-complex structure (i.e., easier to synthesize) while retaining high stability.^11^ These attributes reinforced the potential of cyclic peptide scaffolds to engineer SFTI-1-based ligands for the melanocortin 1 receptor^12^ and the bradykinin B1 receptor,^13^ among other drug candidates.^14^

In light of the ongoing opioid crisis worldwide,^15^ the κ-opioid receptor (KOR) has emerged as an alternative therapeutic target for the development of safer pain medications without deleterious side effects commonly associated with the μ-opioid receptor (MOR).^16^ The KOR belongs to the class of inhibitory GPCRs and is activated by the endogenous peptide ligand dynorphin A (dyn A) 1–17.^17^ Although KOR agonists are effective for pain treatment,^2^ they are frequently associated with adverse effects including sedation, dysphoria, and hallucinations.^18^ Thus, while KOR agonists represent promising analgesics, they cause side effects that limit their therapeutic potential. Recently, a novel paradigm in KOR signaling has emerged, termed as biased signaling, with the hypothesis that ligands favorably activating G protein-dependent signaling pathways over β-arrestin-dependent ones by stabilizing distinct KOR conformations might facilitate the development of safer and more effective pain drugs.^19^ Despite the notion that β-arrestin signaling is required for the development of side effects remains controversial, studies on the MOR have corroborated enhanced and prolonged analgesia in the absence of β-arrestin recruitment.^20,21^

Since KOR-dependent side effects primarily occur by means of its activation in the central nervous system (CNS), targeting the KOR in the periphery constitutes an intriguing strategy to develop analgesic pharmaceuticals devoid of centrally mediated side effects.^22^ For instance, peripherally restricted MOR agonists demonstrated analgesic efficacy in vivo, but rapid development of tolerance limits their therapeutic use,^23^ while δ-opioid receptor (DOR) peripheral agonists exhibit low analgesic efficacy in vivo, possibly due to limited surface expression.^24^ In contrast, peripherally acting KOR agonists exerted analgesic activity in numerous visceral pain models, providing evidence that peripherally restricted KOR agonists may be leveraged to treat several visceral pain conditions, including postoperative, ileus, pancreatitis, and labor pain, and bowel disorders.^25,26^ In fact, difelikefalin (CR845) is a peripherally restricted KOR agonist with limited ability to penetrate the CNS and it has recently been approved for the treatment of postoperative pain.^2^ However, difelikefalin requires intravenous administration and its oral activity is limited, which restrict its potential use as a broad-spectrum analgesic.^27^

Inspired by the traditional use of sunflower (*Helianthus annuus*) preparations for anti-inflammatory and analgesic applications,^28^ we report here the discovery of a cyclic peptide ligand from sunflower seeds targeting the KOR. The SFTI-1 scaffold triggered the design of stable and potent KOR ligands (termed as helianorphins) with low nanomolar affinity and potency, selectivity for the KOR over other opioid receptors, and strong peripheral in vivo analgesic efficacy in a mouse model of visceral pain.

## Results

### Grafting onto SFTI-1 Generates Potent KOR Ligands

Hitherto, peptides with KOR activity have been identified in fungi and vertebrates.^26,29,30^ Recently, the analgesic activity of a methanolic extract of sunflower seeds was reported^28^ and prompted us to investigate whether SFTI-1, one of the main peptides in the extract of sunflower seeds (Figure 1A,B), might be a novel ligand of the KOR. Indeed, radioligand binding studies revealed significant but moderate displacement of [^3^*H*]-diprenorphine with a micromolar concentration of SFTI-1 in HEK293 cell membrane preparations stably expressing the mouse KOR (Figure 1C, Table 1). We then opted to improve the pharmacological profile of SFTI-1 by utilizing “grafting” (Figure 1D), an approach that has been successfully applied to develop other peptidergic GPCR ligands.^12,13^ SFTI-1 is a potent trypsin inhibitor^31^ characterized by backbone cyclization, structurally distinct loops (hereafter referred to as the binding and cyclization loops), and one disulfide bond.^32^ The cyclization loop contains the native aspartic acid–glycine (D–G) cyclization site, and the binding loop incorporates the lysine (K) residue essential for trypsin inhibitory activity. Extensive structure–activity relationship studies of dyn A 1–17 have identified its fragment variants critical for pharmacological activity.^33^ While the N-terminal “message sequence” (YGGF) is essential for binding to all opioid receptors, the C-terminal “address sequence” (LRRIRPKLKWDNQ) is required for potency and KOR selectivity.^34^ Based on published knowledge, we initiated grafting by incorporating dyn A 1–13 and dyn A 1–8 sequences, hereafter referred to as helianorphin-1–4, into both loops of SFTI-1 (Figure 1D, Table 1). The design strategy is illustrated in Figure 1D.

**Table 1:** Pharmacological Data of Helianorphins at the KOR^a^

		affinity	potency/efficacy cAMP	
ligand	sequence*	*K*i ± SD (M)	EC_50_ ± SD (M)	*E*_max_ ± SD (%)
dyn A 1–13	YGGFLRRIRPKLK-NH_2_	3.1 ± 0.3 × 10^–10^	2.8 ± 0.7 × 10^–9^	100
SFTI-1	c-CTKSIPPICFPDGR	>3.0 × 10^–5^	n.d.	n.d.
helianorphin-1	c-CYGGFLRRIRPKLKCFPDGR	2.2 ± 0.6 × 10^–7^	1.7 ± 0.1 × 10^–6^	106 ± 8.4
helianorphin-2	c-CTKSIPPICYGGFLRRIRPKLK	9.7 ± 0.5 × 10^–8^	1.3 ± 0.4 × 10^–6^	102 ± 10
helianorphin-3	c-CYGGFLRRICFPDGR	3.2 ± 0.4 × 10^–6^	1.4 ± 0.3 × 10^–6^	85.1 ± 1.8
helianorphin-4	c-CTKSIPPICYGGFLRRI	2.5 ± 0.05 × 10^–6^	4.7 ± 1.3 × 10^–6^	87.8 ± 33
helianorphin-5	c-CYGGFLRICFPDGR	>10^–5^	n.d.	n.d.
helianorphin-6	c-CTYGGFLRCFPDGR	>10^–5^	n.d.	n.d.
helianorphin-7	c-CT*A*SIPPICYGGFLR	>10^–5^	n.d.	n.d.
helianorphin-8	c-CTYGGFPICFPDGR	>10^–5^	n.d.	n.d.
helianorphin-9	c-CT*A*SIPPICYGGFR	>10^–5^	n.d.	n.d.
helianorphin-10	c-CTfflkPICFPDGR	>10^–5^	n.d.	n.d.
helianorphin-11	c-CT*A*SIPPICfflkR	>10^–5^	n.d.	n.d.
helianorphin-12	c-CTffbrPICFPDGR	2.3 ± 0.5 × 10^–6^	1.5 ± 0.3 × 10^–6^	117 ± 12
helianorphin-13	c-CT*A*SIPPICffbrR	>10^–5^	n.d.	n.d.
helianorphin-14	c-CYGGFLRRIRpKLKCFPDGR	2.2 ± 0.3 × 10^–7^	n.d.	n.d.
helianorphin-15	c-CffbrLRRIRPKLKCFPDGR	1.7 ± 0.1 × 10^–7^	n.d.	n.d.
helianorphin-16	c-CT*A*SIPPICffbrLRRIRPKLK	2.5 ± 0.2 × 10^–7^	n.d.	n.d.
helianorphin-17	c-CT*A*SIPPICffbrLRRIRpKLK	1.2 ± 0.5 × 10^–7^	n.d.	n.d.
helianorphin-18	c-CTYGGFLRCRIRPKLK	6.5 ± 1.5 × 10^–7^	n.d.	n.d.
helianorphin-19	c-CYGGFLRRCIRPKLK	2.1 ± 0.8 × 10^–8^	4.5 ± 0.6 × 10^–8^	108 ± 11

a Data are from three to six independent biological replicates. *Grafted epitopes are underlined; lysine (K) was replaced with alanine (A) to abolish trypsin inhibitory activity and is shown in the italic font; f, l, k, b, and r denote D-enantiomers of phenylalanine, leucine, lysine, norleucine, and arginine, respectively. n.d., not determined; dyn A, dynorphin A; and SFTI, sunflower trypsin inhibitor.

**Figure 2:**
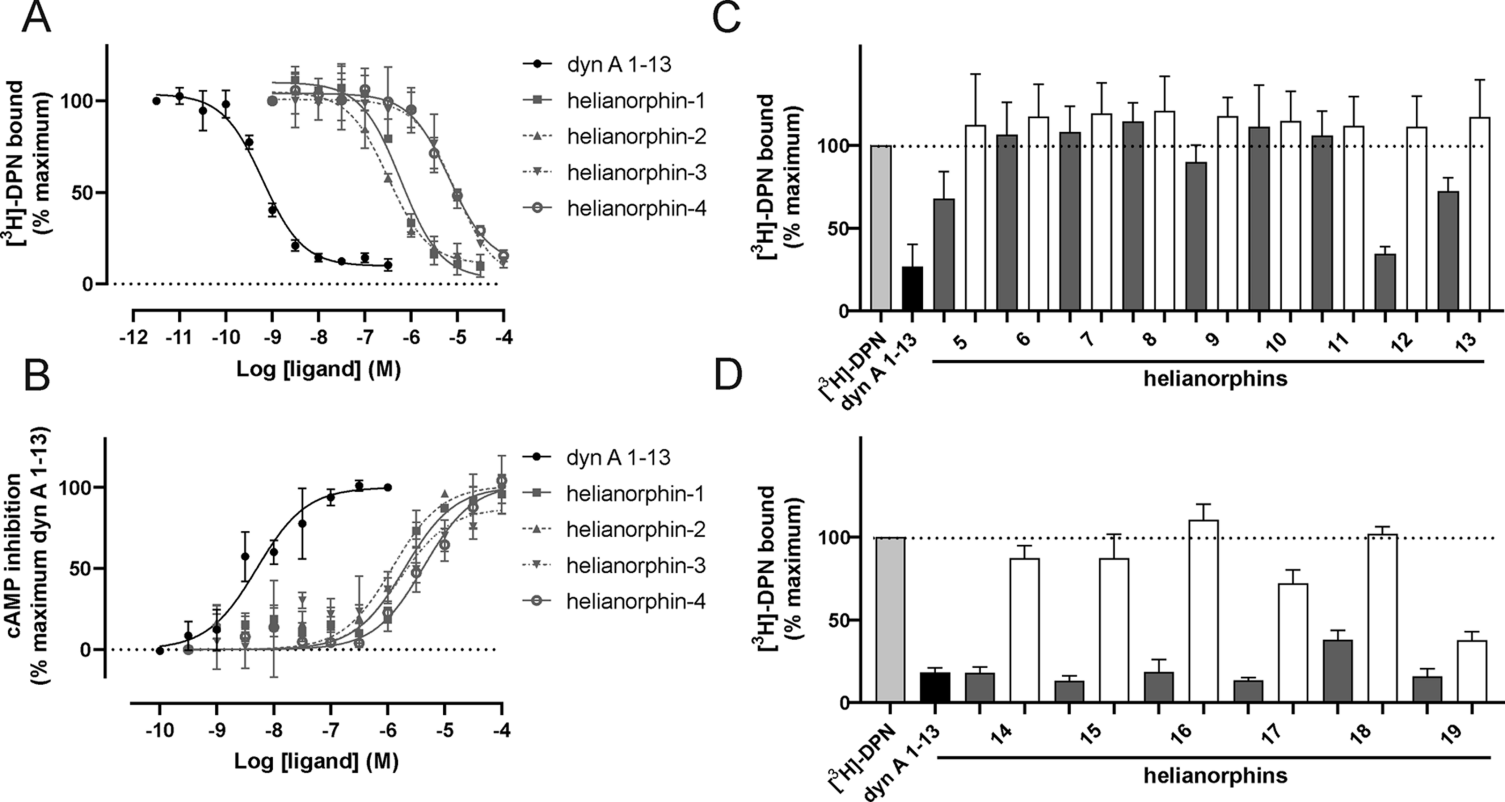
(A) Radioligand displacement of helianorphins 1–4 on HEK293 membrane preparations stably expressing the mouse KOR (*n* = 3). Dynorphin A (dyn A) 1–13 was used as a positive control (*n* = 3). (B) Concentration-dependent cAMP inhibition following receptor activation by helianorphin-1 and -2 (*n* = 3) and helianorphin-3 and -4 (*n* = 4) in HEK293 cells stably expressing the mouse KOR. Dynorphin A (dyn A) 1–13 was used as a positive control (*n* = 3). (C) Two-point radioligand displacement assay of helianorphins 5–13 (*n* = 3) and (D) helianorphins 14–19 (*n* = 5) at the mouse KOR. Radioligand [^3^*H*]-diprenorphine (DPN; 1 nM) with or without 10 μM (dark gray bars) or 100 nM (white bars) of helianorphins was used. Dynorphin A (dyn A) 1–13 was used as a positive control (*n* = 3). Specific binding was obtained by subtraction of nonspecific binding from total binding. Data are presented as mean ± SD and are normalized to the percentage of maximum binding.

The peptides were screened in concentration-dependent radioligand binding assays. Helianorphin-1 and -2 displaced tritiated diprenorphine with *K*_i_ values of 220 and 97 nM, respectively (Figure 2A, Table 1). In contrast, helianorphin-3 and -4 bound to the KOR with low micromolar affinity, that is, *K*_i_ values of 3.2 and 2.5 μM, respectively (Figure 2A, Table 1). Since the KOR is an inhibitory GPCR whose activation results in an inhibition of adenylyl cyclase and thus leads to reduced cellular cAMP levels, the peptides were tested in a functional cAMP assay. Despite nanomolar affinities, helianorphin-1 and -2 fully activated the receptor but only with micromolar potency similar to that of helianorphin-3 and -4 (Figure 2B, Table 1).

**Figure 3:**
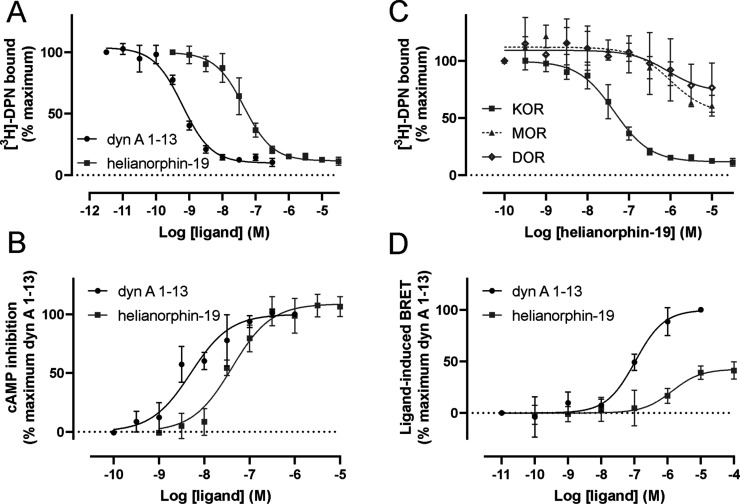
(A) Concentration-dependent competition binding curves of helianorphin-19 (*n* = 3) and dynorphin A (dyn A) 1–13 as a positive control (*n* = 3) using 1 nM [^3^*H*]-diprenorphine (DPN) and mouse KOR containing membrane preparations. The difference between total binding and nonspecific binding is defined as specific binding. Data are mean ± SD. (B) KOR activation induced by helianorphin-19 (*n* = 3) and the positive control dyn A 1–13 (*n* = 3) was measured in a concentration-dependent manner by quantifying cAMP in HEK293 cells stably expressing the mouse KOR. (C) Selectivity profile of helianorphin-19 was determined in radioligand displacement studies on HEK293 cell membranes stably expressing the mouse μ-opioid receptor (MOR) and δ-opioid receptor (DOR) (*n* = 2). Binding of helianorphin-19 on mouse KOR cells is included for comparison. (D) β-arrestin-2 recruitment assay of helianorphin-19 and dyn A 1–13 was conducted in HEK293 cells transiently expressing mouse KOR-EGFP and β-arrestin-2-nano-luciferase and using furimazine as an enzyme substrate. Data are mean ± SD and are normalized to the percentage of maximum recruitement of dyn A 1–13 (*n* = 5). The EC_50_ values for helianorphin-19 and dyn A 1–13 were 1.4 ± 0.3 μM and 116 ± 39 nM, respectively. The *E*_max_ value for helianorphin-19 was 41.1 ± 3.7%. EGFP—enhanced green fluorescence protein. BRET—bioluminescence resonance energy transfer.

To examine whether the size and sequence of epitopes or stereochemistry of certain residues affects pharmacological properties at the KOR and hence to further improve affinity and potency of the nature-derived peptide scaffold SFTI-1, we grafted dyn A 1–6 and dyn A 1–4 and modified tetrapeptide sequences of the approved peptide drug difelikefalin (CR845)^35,36^ (Table 1). Regardless of the epitope sequences, the lysine residue (K) in the binding loop was replaced with alanine (A) to eliminate trypsin inhibitory activity of SFTI-1.^37^ These peptides were examined in KOR binding experiments via two-point radioligand displacement studies (Figure 2C, Table 1). Grafting hexa- and tetrapeptides onto SFTI-1 did not improve binding affinity at the KOR (Figure 3C, Table 1). These data are in line with previous structure–activity studies of dyn A 1–13, in that removal of seven or nine amino acids at the C-terminus reduces its affinity.^34^ Helianorphins containing the bioactive epitope with d-amino acids, that is, 2× phenylalanine (f), norleucine (b), and arginine (r), in the binding loop showed the most pronounced binding effect (Figure 2C, Table 1). A detailed pharmacological analysis of helianorphin-12 revealed that the peptide binds to and fully activates the KOR in a concentration-dependent manner with a *K*_i_ of 2.4 μM and an EC_50_ of 1.4 μM, respectively (Table 1, Figure S1B, Supporting Information).

### Helianorphin-19 Is a Selective KOR Ligand

Given that helianorphins comprising dyn A 1–13 displayed the most promising binding affinities at the KOR, we further modified this epitope (Table 1). We incorporated modifications that previously enhanced affinity of dyn A 1–13,^38^ and we substituted the N-terminal YGGF domain of dyn A 1–13 with the most active tetrapeptide from the previous series of synthesized helianorphins (Table 1). Moreover, we designed helianorphins by dividing dyn A 1–13 into two fragments and placing them in either the binding or cyclization loop of SFTI-1 while retaining its backbone cyclic structure and disulfide bond orientation (Figure 1D, Table 1). The ability of peptides to displace a radioligand was measured in a two-point binding assay (Figure 2D, Table 1). As expected, helianorphins with modified dyn A 1–13 sequences demonstrated increased binding toward the KOR with helianorphin-19 being the most promising candidate (Figure 2D, Table 1). Affinity measurements and cAMP concentration–response curves exhibited distinct *K*_i_ and EC_50_ values of these ligands (Figure 3A, Table 1, Figure S1A, Supporting Information). Specifically, helianorphin-19 bound to the KOR with an affinity of 21 nM (Figure 3A, Table 1, Figure S1A, Supporting Information). Helianorphin-19 activated the KOR with an EC_50_ of 45 nM and *E*_max_ of 108% (Figure 3B, Table 1). This yielded helianorphin-19 as a lead agonist for further studies. To assess receptor selectivity, the affinity of helianorphin-19 was determined in HEK293 membranes stably expressing the mouse MOR and DOR. Helianorphin-19 partially displaced tritiated diprenorphine from MOR- and DOR-binding sites with *K*_i_ values >10 μM, which exemplifies a more than 200-fold enhanced selectivity for the KOR (Figure 3C).

### Helianorphin-19 Preferentially Modulates the G Protein Pathway over that of β-Arrestin-2

Driven by these findings including that helianorphin-19 is a KOR-selective agonist with nanomolar affinity and potency, it was interesting to determine the ability of helianorphin-19 to induce β-arrestin-2 (also known as arrestin-3) recruitment in a bioluminescence resonance energy transfer (BRET) assay (Figure 3D). β-arrestins are cytosolic molecules, which bind to the activated and GPCR kinase (GRK)-phosphorylated KOR, leading to its desensitization, internalization via a clathrin- and dynamin-dependent pathway, recycling to the plasma membrane or lysosomal degradation.^39^ A growing body of the literature has linked β-arrestins to prolonged analgesia and severe side effects, underscoring the potential of G protein-biased ligands to develop analgesics without side effects associated with classic opioids.^19,40^ Using KOR-EGFP and β-arrestin-2 nano-luciferase constructs, we conducted a BRET assay to explore β-arrestin-2 recruitment. Compared to the reference ligand dyn A 1–13 which fully recruited β-arrestin-2 with an EC_50_ of 116 nM, helianorphin-19 only partially recruited β-arrestin-2 with an *E*_max_ of 41% and an EC_50_ of 1.4 μM (Figure 3D). These findings indicate that helianorphin-19 exhibits impaired β-arrestin-2 recruitment and might thus trigger enhanced and prolonged analgesic activity compared to dyn A 1–13.

### Helianorphin-19 Is Stable in the Gastrointestinal Tract

To demonstrate the potential of our lead peptide to be active via the enteric route of administration, we determined the stability of helianorphin-19 in simulated gastric fluid (SGF). The prolonged half-life of helianorphin-19 (*t*_1/2_ = 3.1 h) versus that of dyn A 1–13 (fully degraded after 15 min) suggested its stability and capability of surviving under conditions in the gastrointestinal tract (Figure S2, Supporting Information). To confirm the stability of helianorphin-19 in vitro, we determined its efficacy after intracolonic administration in a mouse model of abdominal pain.

### Helianorphin-19 is Active in the CVH Mouse Model of Abdominal Pain

**Figure 4:**
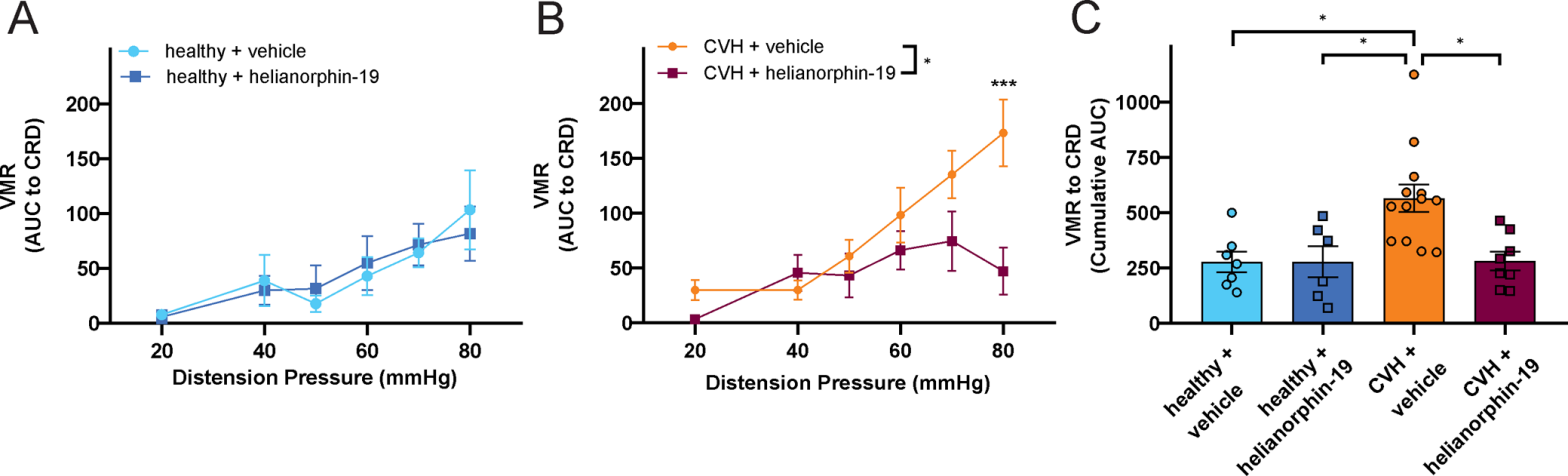
(A) In healthy control mice, intracolonic administration of helianorphin-19 (10 μM, 100 μL of bolus) had no significant effect on VMRs to CRD compared with vehicle treatment (healthy + vehicle: *N* = 7 vs healthy + helianorphin-19: *N* = 6; non-significant, *P* > 0.05, 2-way RM ANOVA with Sidak’s multiple-comparison test). (B) In mice with CVH, intracolonic helianorphin-19 (10 μM, 100 μL bolus) significantly reduced VMRs to CRD, particularly at 80 mmHg CRD (CVH + vehicle: *N* = 13 vs CVH + helianorphin-19: *N* = 8; *P < 0.05, 2-way RM ANOVA with ***P < 0.001, Sidak’s multiple-comparison test). (C) VMR to CRD data from healthy and CVH mice treated with either vehicle or helianorphin-19 represented as the cumulative area under the curve (cumulative AUC, each dot represents data from an individual mouse in each cohort). Overall, CVH mice treated with a vehicle had significantly greater VMRs to CRD compared with healthy control mice treated with either a vehicle (*P < 0.05) or helianorphin-19 (*P < 0.05). CVH mice treated with helianorphin-19 (10 μM, 100 μL bolus) display significantly reduced VMRs to CRD compared with vehicle-treated CVH mice (*P < 0.05). Analysis based on Kruskal–Wallis test followed by Dunn’s multiple-comparison test.

Previous studies unveiled functional KOR upregulation during chronic visceral hypersensitivity (CVH) associated with chronic abdominal pain.^25^ Thus, we assessed the capability of helianorphin-19 to modify colonic nociception in vivo in mice under healthy and CVH conditions (Figure 4A–C). To do this, we recorded the visceromotor response (VMR) across a variety of colorectal distension (CRD) pressures.^41−44^ The VMR to CRD has previously provided an invaluable way of determining pro- and anti-nociceptive mechanisms associated with CVH. Here, we show that a single intracolonic dose of helianorphin-19 had no effect on the VMR to CRD in healthy naïve mice (Figure 4A). In contrast, the helianorphin lead peptide significantly reduced the VMR to CRD in CVH mice (Figure 4B,C). The observed effect is consistent with previously published data using asimadoline and conopeptide-derived analogue 39, which were only active in the CVH state and induced antinociceptive effects by activating the KOR expressed on the peripheral endings of colonic nociceptors.^26,45^

**Figure 5:**
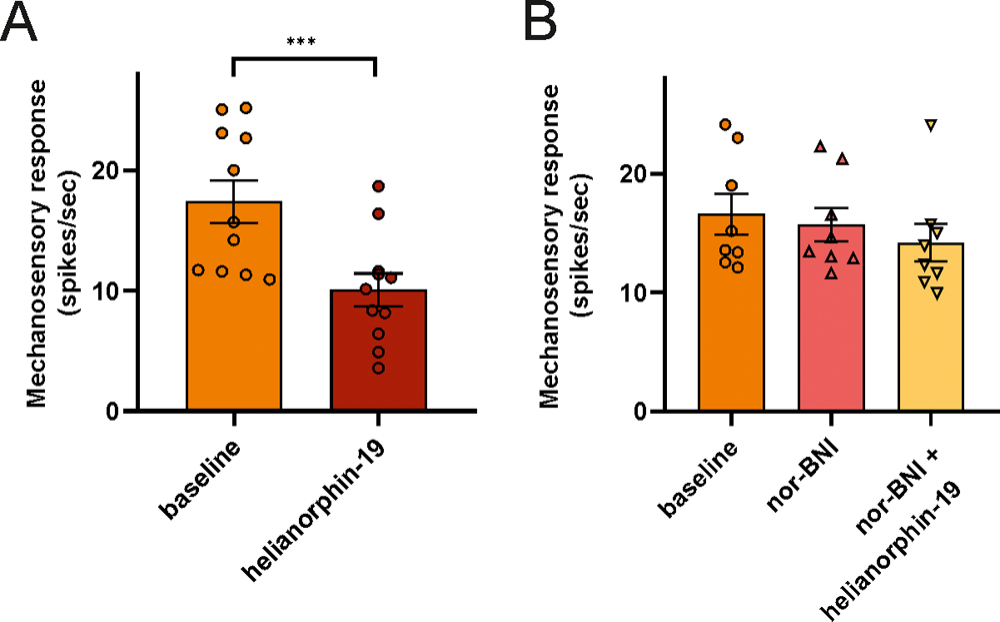
(A) Application (5 min) of helianorphin-19 (1 μM) to colonic nociceptor endings from CVH mice caused a decrease in action potential firing to mechanical stimulation ex vivo (*n* = 11 nociceptors; ***P < 0.001, paired *t*-test). (B) Pre-incubation (5 min) of colonic nociceptor endings with the selective KOR antagonist norbinaltorphimine (nor-BNI; 0.1 μM) did not affect nociceptor mechanosensitivity. However, nor-BNI did block helianorphin-19 (1 μM)-induced inhibition of colonic nociceptor action potential firing to mechanical stimuli ex vivo (*n* = 8 nociceptor; *P* > 0.05; one-way ANOVA followed by Bonferroni post hoc tests).

We then investigated whether the in vivo analgesic effect of helianorphin-19 was indeed mediated by activation of KORs expressed by sensory afferent neurons innervating the colon of CVH mice. To do this, we performed single-unit extracellular recordings from splanchnic nerves, using an ex vivo preparation, as previously described.^45,46^ We first determined whether helianorphin-19 was able to reduce the response of colonic nociceptors to mechanical stimulation with calibrated von Frey filaments (2 g vhf). Helianorphin-19 application significantly reduced action potential firing of colonic nociceptors evoked by mechanical stimulation (Figure 5A). To confirm whether the helianorphin-19 effect was mediated by the KOR, we then examined whether helianorphin-19 was able to reduce colonic nociceptor mechanosensitivity when applied in conjunction with norbinaltorphimine (nor-BNI), a selective KOR antagonist. Nor-BNI application did not affect CVH colonic nociceptor responses to mechanical stimuli (Figure 5B). Importantly, nor-BNI also prevented helianorphin-19-induced inhibition of colonic nociceptor mechanosensitivity.

### Helianorphin-19 Does Not Induce Motor Coordination and Central Analgesia

**Figure 6:**
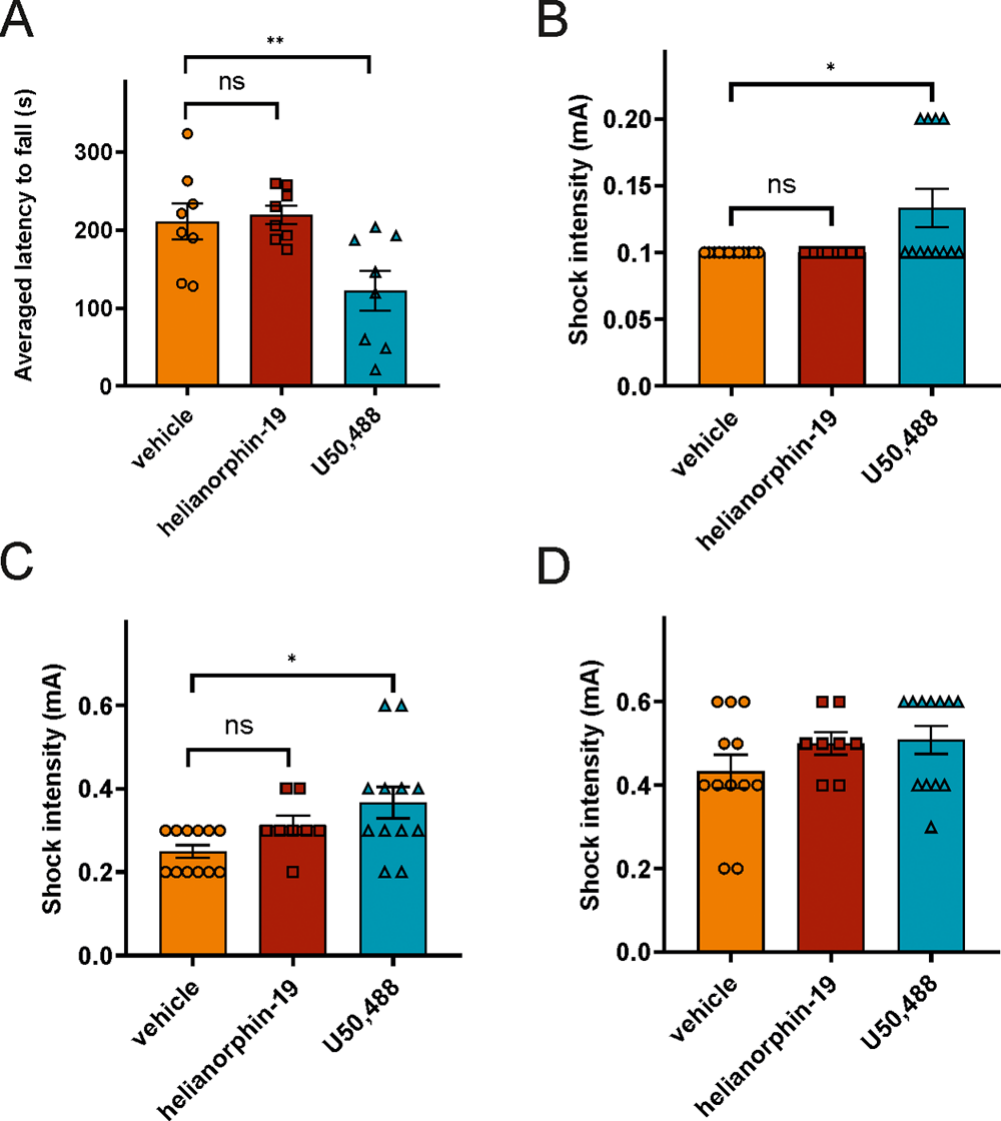
(A) Averaged latency to fall from the rotarod(s). (B) Shock intensity at which mice exhibited their first flinch response. (C) Shock intensity at which mice exhibited their first vocalization. (D) Shock intensity at which mice exhibited their first jump. Mice were treated with a single dose of helianorphin-19 (5 mg/kg) and U50,488 (5 mg/kg). All data are presented as mean ± SEM. Statistical significances resulting from one-way ANOVA followed by Fischer’s LSD test are displayed. *p < 0.05, **p < 0.01, *N* = 8–12/group.

To exclude that the analgesic effects of helianorphin-19 are due to penetration of the CNS, we measured whether helianorphin-19 alters motor coordination in the rotarod test (as a model for central sedation). Herein, helianorphin-19 did not impair rotarod performance compared to the selective KOR agonist U50,488, which elicited significant deficits in motor coordination^47^ (Figure 6A). To further support the peripherally restricted action of helianorphin-19, we used the jump-flinch test to ascertain its sensitivity for central pain. The jump-flinch test has been previously described as an alternative to the tail-flick and hot plate assays to examine morphine-induced central analgesia.^48,49^ Herein, we monitored three behaviors of mice, namely, flinch, vocalization, and jump. In flinch and vocalization behaviors, U50,488 significantly increased shock intensity compared to helianorphin-19 (Figure 6B,C). However, mice jumped at similar intensity upon administration of helianorphin-19 and U50,488 when compared to those with a vehicle (Figure 6D). Taken together, these findings highlight the potential of the nature-derived cyclic peptide scaffolds to design and develop novel peripheral KOR analgesics.

## Discussion and Conclusions

Opioids have been the focus of intensive research for decades aimed at developing safer and more effective pain treatments.^50^ MOR agonists, including morphine and fentanyl, constitute the mainstay of pain management, yet the therapeutic use of these medications often results in deleterious side effects including respiratory depression, sedation, nausea, constipation, tolerance, and dependence.^51^ As a result, the prescription of opioid pain relievers has recently escalated into an opioid crisis, leading to addiction and thousands of overdose deaths in North America, Europe, and Australia/New Zealand.^15^ Thus, there is an unmet clinical need to develop analgesics with improved therapeutic profiles.

Inspired by recent studies on the analgesic activity of a methanolic extract of sunflower seeds (*H. annuus*),^28^ we explored the analgesic potential of SFTI-1, a cyclic peptide expressed by sunflower seeds. Leveraging its intrinsic biostability and significant binding at the KOR—an opioid receptor with increasing attention as a potential therapeutic target for the treatment of visceral pain—helianorphin peptides were designed by grafting dyn A and difelikefalin-like analogues onto the SFTI-1 scaffold. This concept has yielded helianorphin-19, a full agonist of the KOR with nanomolar affinity and potency (Table 1). Interestingly, helianorphin-19 displayed approximately 200-fold increased selectivity for the KOR over the MOR and DOR. This agrees with recent studies, which led to the development of potent SFTI-1-based peptide ligands with improved selectivity for the melanocortin 1 receptor.^12^

KOR agonists lack the side effects usually observed for their MOR counterpart. However, centrally associated side effects including sedation, dysphoria, and hallucinations deter their therapeutic use.^18^ Seminal studies demonstrating enhanced morphine analgesia and mitigated side effects in the absence of β-arrestin-2 in mice^20^ initiated efforts to design biased ligands preferentially activating G protein-mediated over β-arrestin-dependent pathways with the aim to develop safer KOR therapeutics devoid of adverse effects common for KOR modulation in the CNS.^50^ However, biased signaling is a timely but controversial theme in the field of opioid receptors. For instance, despite the recent approval of the first biased MOR ligand (oliceridine) by the USA Food and Drug Administration for intravenous use, concerns have been raised which question the concept of β-arrestin pathways’ contribution to harmful side effects.^21^ Oliceridine displayed promising preclinical in vivo findings,^52^ yet its clinical evaluation revealed a safety profile similar to that of conventional opioids.^53^ It exhibited respiratory depression, nausea, vomiting, and adverse gastrointestinal side effects, not significantly distinct from morphine.^54,55^ Furthermore, while Manglik et al. identified a promising G protein-biased agonist, PZM21, to be devoid of respiratory depression,^56^ Hill and colleagues re-examined the signaling profile of PZM21 and observed that it induces respiratory depression in a manner similar to morphine.^57^ Morphine- and fentanyl-mediated side effects, including respiratory depression and constipation, have been further demonstrated in a knock-in mouse expressing a phospho-deficient MOR mutant unable to recruit β-arrestins^21^ and in a β-arrestin-2 knockout mouse.^58^ The evidence that canonical G protein signaling of the MOR contributes to respiratory depression and constipation has been additionally confirmed by other studies.^59,60^ Recently, Gillis et al. unraveled that the low intrinsic efficacy of G protein-biased MOR agonists may explain their improved side effect profiles rather than their ability to minimally recruit β-arrestin.^61^ Consequently, constraining endomorphin-1 using β,α-hybrid dipeptide/heterocycle scaffolds, De Marco and colleagues developed a stable KOR partial agonist with in vivo antinociception and possibly reduced side effects.^62^ However, hitherto, several studies demonstrated that the lack of β-arrestin signaling leads to enhanced and prolonged analgesia.^20,21,56^ Accordingly, the preference of helianorphin-19 for the G protein-stimulated pathway (full agonist, EC_50_ = 45 nM) over β-arrestin-2 recruitment (partial agonist, EC_50_ = 1.4 μM) allows us to speculate that helianorphin-19 may display greater and prolonged duration of peripheral antinociception in vivo. Importantly, a recent study provides evidence that ligands with lowered efficacy in the arrestin pathway are sufficient for dissociating the antinociceptive effects from centrally mediated KOR adverse effects; two G protein-biased agonists (HS665 and HS666) that partially recruit β-arrestin-2 are potent antinociceptive molecules with reduced sedation and absence of conditioned place aversion.^63^ Similarly, a G protein-biased cyclic peptide KOR agonist, LOR17, showed no significant β-arrestin-2 recruitment, while it elicited potent analgesia in vivo without promoting centrally mediated side effects.^64^

The roles of β-arrestins in the development of peripherally mediated KOR side effects have been neglected so far. Previous evidence indicates that peripheral MOR signaling gives rise to the development of morphine tolerance and opioid-induced hyperalgesia and constipation, possibly involving the interaction with β-arrestins.^23,52^ Although intravenous use of difelikefalin is not associated with the development of tolerance and constipation,^27^ the role of β-arrestins in the peripheral KOR signaling warrants further investigation. In this regard, peripherally mediated KOR agonists hold promise to develop safer and more effective analgesics. Snyder et al. broadened our understanding of peripheral KOR-dependent analgesia by identifying specific types of somatosensory neurons expressing the KOR and providing a rationale of peripherally restricted KOR agonists for pain treatment.^22^ To address the therapeutic relevance of helianorphin-19, we ascertained its ability to modify colonic nociceptor mechanosensitivity in a mouse model of abdominal pain. An intracolonic administration of helianorphin-19 revealed a significant decrease in VMR to CRD in mice associated with CVH, whereas no significant change to the vehicle was observed in healthy mice. Moreover, in ex vivo preparations of the mouse colon with attached sensory nerves, helianorpin-19 significantly reduced CVH colonic nociceptor firing evoked by mechanical stimuli. The ex vivo analgesic effects of helianorphin-19 were revoked by pre-treatment with the specific KOR antagonist nor-BNI. These findings are consistent with previous findings obtained using other KOR agonists.^25,26^ We chose to perform antagonist treatment in the ex vivo model, since nor-BNI is capable of crossing the blood–brain barrier in vivo.^65^ Although the ex vivo findings demonstrate the involvement of the KOR in analgesic activity in the colon, we cannot exclude the possibility that the KOR (or other receptors) located elsewhere might contribute to the analgesic efficacy observed in vivo.

Furthermore, these data validate the SFTI-1 framework as a platform for development of peripherally active KOR agonists and substantiate previous in vivo findings of asimadoline and conorphin-derived compound 39.^26,45^ Enadoline (CI-977) and spiradoline (U-62066E) are prototypic small-molecule KOR agonists, which failed in clinical trials due to central side effects associated with significant blood–brain barrier penetration.^27^ In contrast, the peripherally restricted KOR agonist asimadoline attenuated pain response following colonic distension with the greatest efficacy against symptoms in patients of diarrhea-predominant irritable bowel syndrome.^66^ However, asimadoline induced hyperalgesia in non-visceral postoperative pain.^67^ Owing to their size and common hydrophilic nature, peptides are suitable for reducing CNS penetration while maintaining peripheral efficacy. Accordingly, we demonstrated that helianorphin-19 displays reduced CNS penetration, as it did not impair motor coordination or induce central analgesia in vivo. However, since only a single dose (5 mg/kg) of helianorphin-19 was measured in the rotarod and jump-flinch assays, it is recommended to (i) establish a dose–response relationship of helianorphin-19 in several assays in future studies (including hot-plate and/or tail-flick assays) and (ii) perform a detailed pharmacokinetic study to ultimately confirm its peripheral restriction. In fact, difelikefalin (CR845) is an all-d-amino acid tetrapeptide developed by Cara Therapeutics to treat abdominal pain after abdominal surgery, by selectively targeting the peripherally expressed KOR.^2^ Although difelikefalin (CR845) is devoid of detrimental effects linked to the centrally expressed KOR, its intravenous delivery remains a key obstacle for its widespread use. In this study, we have provided evidence that the cyclic, disulfide-stabilized peptide helianorphin-19 is active via intracolonic administration and significantly more resistant against conditions in the gastrointestinal tract, suggesting that the SFTI-1 scaffold is ideally suited to design and develop potent and efficacious KOR ligands to be delivered by the enteric route. The stability of helianorphin-19 is further supported by recent study of Tam and colleagues, who designed a SFTI-1-based bradykinin B1 receptor antagonist with high stability under acidic conditions in the stomach and significant analgesic activity in an animal pain model upon oral application;^13^ whether helianorphin-19 is orally active warrants further investigations. Furthermore, grafting onto the SFTI-1 scaffold has facilitated the development of cyclic peptides stable in human serum and active in a colitis-induced mouse model of inflammatory bowel disease.^68^ Enteric delivery remains the most desirable route of drug administration, in particular for colitis and inflammatory bowel disease,^69^ and the cyclic plant-derived peptides are a promising class of molecules, which may energize design and development of compounds with enteric applications.

In conclusion, we describe the first plant-derived cyclic opioid-like peptide with moderate binding toward the KOR. The observed affinity of SFTI-1 stimulated design and development of helianorphin-19, a potent KOR agonist with decreased β-arrestin-2 recruitment and analgesic activity in an animal model of abdominal pain. Its efficacy via the enteric route of administration exemplifies the potential of helianorphin peptides as an intriguing class of molecules to design and develop stable peptide analgesics targeting the peripherally expressed KOR for the treatment of visceral pain. In light of the ongoing opioid crisis,^15^ there is an urgent need for novel pain medication. Current small-molecule drugs such as morphine or fentanyl are associated with severe and sometimes fatal side effects.^51^ The quest for the next-generation analgesics for safer pain treatment continues to be an active area of scientific research. In this context, nature-derived peptides are a rich source for drug discovery and development, since they occupy a chemical space that is not easily represented by small molecules.^5^ Peptides derived from toxic plants^70^ and venomous animals including cone snails, spiders, and scorpions^71^ have proven to be particularly attractive pharmacological research tools to study the physiology of pain and lead molecules for the development of analgesic drugs by targeting ion channels.

Nature-derived peptides embrace a growing niche for GPCR ligand discovery.^5^ GPCRs are highly abundant receptors and intriguing molecular targets involved in pain^72^ and other diseases.^1−3^ At the more general level, our study provides guidance on how to utilize stabilized plant peptide scaffolds for molecular grafting of endogenous neuropeptides and peptide hormones to develop optimized pharmacological probes with enhanced receptor subtype selectivity and pathway specificity. At the very least, we present the basis for exploring plants as a valuable repository for the discovery of stable peptide ligands targeting GPCRs associated with abdominal analgesia.

## Experimental Section

### Materials

Dyn A 1–13 amide trifluoroacetate salt was from Bachem (Austria). Naloxone and (±)-*trans*-U50,488-methanesulfonate salt were obtained from Sigma (Austria). [^3^*H*]-diprenorphine (34.6 Ci/mmol) was from PerkinElmer (Austria), and the cAMP G_i_ kit was from Cisbio (Germany). The jetPRIME transfection reagent was from Polyplus (Austria).

### Animals and Ethics

Male C57BL/6J mice were used in all experiments. All animals used for studying abdominal pain were sourced from the specific and opportunistic pathogen-free facility at Animal Bioresources at the South Australian Health and Medical Research Institute (SAHMRI), Adelaide (Office of Gene Technology Regulator and Physical Containment level 2 accredited; certification number, 3767), which is also approved to breed and rederive lines. Mice were originally purchased from The Jackson Laboratory (stock number, 000664; breeding barn MP14; Bar Harbor, ME, USA). Experimental procedures for studying abdominal pain followed the guidelines of the Animal Ethics Committees of the SAHMRI and Flinders University and ethical approval (ethics project SAM195). Male C57BL/6N mice (8 weeks of age) for rotarod and flinch-jump tests were purchased from Charles Rivers (Sulzfeld, Germany). Behavioral experiments were conducted in grouped and housed mice (four mice per cage). Mice were kept at the local animal facility under standard laboratory conditions with a 12:12 h light/dark cycle. Food and water were available ad libitum. The experiments were conducted in agreement with the ARRIVE guidelines and the U.K. Animals (Scientific Procedures Act, 1986 and associated guidelines, EU Directive 2010/63/EU for animal experiments) and approved by the Austrian national ethical committee on animal care and use.

### Plant Extraction

The extract of *H. annuus* (Alfred Galke GmbH, Germany) and peptide-enriched fractions have been prepared as previously described.^73^

### Peptide Synthesis and Characterization

All peptides were synthesized on an automated peptide synthesizer using fluorenylmethoxycarbonyl (Fmoc) chemistry as previously described,^74^ using 2-chlorotrityl resin, 2-(6-chloro-1*H*-benzo-triazol-1-yl)-1,1,3,3-tetramethylaminium hexafluorophosphate, as a coupling reagent and standard amino acids as listed in Cheneval et al.^74^ Backbone cyclization of peptides was carried out according to previously published protocols.^12,74^ Briefly, in the first step, peptides were cleaved off the resin with 3 mL of 1% trifluoroacetic acid in dichloromethane (10 times for 5 min). Crude side chain-protected peptides were dissolved in dimethylformamide at a concentration of 2 mM, and cyclization was initiated through addition of 5 mM 2-(7-aza-1*H*-benzotriazole-1-yl)-1,1,3,3-tetramethyluronium hexafluoro-phosphate and 10 mM diisopropylethylamine for at least 3 h at room temperature. Finally, deprotection of side chain-protected amino acids was performed using 10 mL of cleavage cocktail mixture consisting of trifluoroacetic acid/triisopropylsilane/water (95/2.5/2.5, v/v/v) for 2.5–3 h at room temperature. Cyclic reduced peptides were purified by reversed-phase high-performance liquid chromatography (RP-HPLC) on preparative or semipreparative Phenomenex Jupiter C_18_ columns (5 μm, 300 Å, 250 × 21.2 mm or 250 × 10 mm) using a linear gradient from 5 to 65% solvent B (90% acetonitrile, 10% H_2_O, 0.05% trifluoroacetic acid) and flow rates of 8 or 3 mL/min, respectively. Fractions were automatically collected and analyzed by electrospray ionization mass spectrometry (ESI-MS) and either analytical RP-UPLC on a Phenomenex Luna Omega column (1.6 μm C_18_ 100 Å, 50 × 2.1 mm) or RP-HPLC using a Phenomenex Jupiter C_18_ column (5 μM 300 Å, 50 × 2 mm). Oxidation of peptides was carried out by dissolving peptides (1 mg/mL) in an iodine solution (1 mg/mL) in 80% methanol. The reaction was quenched by adding ascorbic acid (10 mg/mL) until discoloration of the iodine. The reaction mixture was subsequently purified by RP-HPLC, and collected fractions were analyzed as described above. Correct mass and purity of all peptides were assessed by ESI- and/or MALDI-MS and analytical HPLC (Table S1, Figure S3A,B Supporting Information).

### NMR

One-dimensional ^1^H and ^13^C NMR spectra were recorded on a Bruker AVANCE 600 MHz at 298 K, with peptides (1–2 mg) dissolved in 90% H_2_O/10% D_2_O. One-dimensional ^1^H NMR spectra were acquired for all helianorphins except for helianorphin-2, while for the helianorphin-19, an additional ^13^C NMR spectrum was acquired (Figures S6–S22 Supporting Information).

### Cell Culture, Transfection, and Cloning

HEK293 cells were cultured in Dulbecco’s modified Eagle medium (DMEM) containing 10% fetal bovine serum and 50 U/mL penicillin and streptomycin and were grown at 37 °C and 5% CO_2_. Cell transfection using 2 μg of plasmids expressing the mouse KOR tagged with EGFP and human β-arrestin-2 fused to nano-luciferase was performed with the jetPRIME transfection reagent as per the manufacturer’s protocol (Polyplus). The mouse KOR was N-terminally cloned into the pEGFP-N1 vector using BamHI and HindIII restriction sites. The HEK293 cell line stably expressing mouse KOR-EGFP was generated by geneticin disulfate (0.8 mg/mL of G418, ROTH, Austria) selection and flow cytometry to select cells for stable cell line propagation. Positive clones were identified by radioligand binding studies.

### Radioligand Competition Binding Assays

Membranes were prepared from HEK293 cells stably expressing the KOR, MOR, and DOR, as previously described.^75^ Radioligand binding studies were carried out in duplicate using standard binding buffer containing 50 mM Tris–HCl (pH 7.4), 10 mM MgCl_2_, and 0.1% BSA. Saturation binding assays with 0.03–10 nM [^3^*H*]-diprenorphine were performed in standard binding buffer on HEK293 cells stably expressing the mouse KOR to determine the equilibrium dissociation constant (*K*_d_) and maximum density of receptors (*B*_max_), whereas 10 μM naloxone was used for nonspecific binding. *K*_d_ and *B*_max_ for the mouse KOR were 0.87 ± 0.06 nM (*n* = 2) and 7166 ± 147 fmol ligand bound per milligram of membrane (*n* = 2), respectively. For competition binding, 75 μL each of [^3^*H*]-diprenorphine (1 nM final), peptide solution (4×), and membrane preparations (7 μg/assay) was incubated in the standard binding buffer, and both saturation and competition binding reactions were incubated for 1 h at 37 °C. Termination of reactions was performed by rapid filtration onto a 0.1% polyethylenimine-soaked GF/C glass fiber filter with a Skatron cell harvester. Competition binding studies with HEK293 cells stably expressing the mouse MOR and DOR were performed with 50 and 20 μg of membranes, respectively.

### cAMP Assay

Functional cAMP assay was conducted in triplicate using HEK293 cells stably expressing the mouse KOR according to the manufacturer’s protocol with slight modifications. Briefly, 2000 cells per 5 μL per well were seeded into a white 384-well plate and incubated with 5 μL of logarithmically spaced concentrations of peptide solutions prepared (2×) in 1× stimulation buffer and forskolin (10 μM final). The reaction mixture was incubated at 37 °C for 30 min followed by addition of Europium cryptate-labeled cAMP and cAMP d2-labeled antibodies (5 μL of each). After incubation for 1 h at room temperature, cAMP quantification was measured by homogenous time-resolved fluorescence resonance energy transfer on a Flexstation 3 (Molecular Devices, San Jose, USA) using a ratio of 665/620 nm.

### BRET Assay

To measure β-arrestin-2 recruitment, HEK293 cells were co-transfected with plasmids transiently expressing human β-arrestin-2 nano-luciferase and mouse KOR-EGFP in 1:10 ratio. After at least 16 h, transfected cells were plated in white clear-bottom cell culture plates in phenol red-free DMEM supplemented with 10% FBS at a density of 50,000 cells in 100 μL per well and allowed to adhere overnight. On the next day, the cells were serum-starved for 1 h at 37 °C in phenol red-free DMEM. Furimazine (Promega, Madison, USA), diluted 1:50, and peptide concentrations were prepared (4×) in Hank’s balanced salt solution and in triplicate. Furimazine was added to the cells and incubated for 5 min at 37 °C followed by measuring the baseline for 5 min. After the addition of peptide ligands and their incubation at 37 °C for 5 min, plates were read for both luminescence at 460 nm for nano luciferase and fluorescence at 510 nm for EGFP using the Flexstation 3 (Molecular Devices, San Jose, USA).

### Peptide Stability in Simulated Gastric Fluid

SGF was prepared according to United States Pharmacopeia specifications (Test Solutions, United States Pharmacopeia 35, NF 30, 2012). For SGF, sodium chloride (0.2 g) was added to a 100 mL flask and dissolved in 50 mL of water. A volume of 0.7 mL of 10 M HCl was added to adjust the pH of the solution to 1.2. To this, 0.32 g of pepsin (Sigma-Aldrich, P7125) was added and dissolved with gentle shaking, and the volume was made up to 100 mL with water. Pepsin was added only after the pH was adjusted to 1.2. The stability of peptides in SGF was tested by adding 5 μL of peptide stock solution (2 mM in water) to 95 μL of SGF followed by incubation at 37 °C. Aliquots (10 μL) were taken at different time points (0 min, 15 min, 30 min, 1 h, 3 h, and 6 h) and added to ice-cold stop reagent (30 μL, methanol for gastric fluid) to inactivate the enzymes. Samples were centrifuged, and the supernatant was analyzed by UPLC–MS using a linear gradient from 1 to 41% solvent B (with 0.1% formic acid) in 40 min and a flow rate of 0.4 mL/min. The peak areas of the peptide were integrated, and the percentage of the peptide left was plotted against the time points indicated above and compared to the initial time point 0. The observed data were fitted to an exponential decay curve with GraphPad Prism software.

### Data Analysis

Data were analyzed using GraphPad Prism (GraphPad Software, San Diego), and statistical analysis was performed by an unpaired *t*-test or ANOVA. Concentration response curves of functional assays were fitted to three-parameter non-linear regression curves with a bottom constrained to zero, a slope of one, and sigmoidal shape at the logarithmic scale to obtain potency (EC_50_) and maximum efficacy (*E*_max_). Graphs were normalized to 100%, which defines the highest concentration of the positive control dyn A 1–13 used in the assay. IC_50_ values from radioligand binding studies were calculated by fitting the data to a three-parameter logistic Hill equation. Inhibition constants (*K*_i_) were observed from IC_50_ values using the approximation developed by Cheng and Prusoff. *K*_d_ values for the mouse MOR and DOR were taken from the literature, being 0.81 ± 0.07 and 1.7 ± 0.2 nM, respectively.^19,76^ Data were normalized to specific binding of [^3^*H*]-diprenorphine in the absence of peptides as maximum percentage (100%), which refers to an average of 4000–5000 fmol/mg for the KOR, 500–1000 fmol/mg for the MOR, and 2000–3000 fmol/mg for the DOR.

### Model of CVH

All animal experiments performed in the manuscript were conducted in compliance with institutional guidelines. Colitis was induced by administration of dinitrobenzene sulfonic acid (DNBS). Briefly, 13-week-old anesthetized female mice were administered an intra-colonic enema of 0.1 mL of DNBS (6 mg per mouse in 30% ethanol) via a polyethylene catheter. Mice were allowed to recover for 28 days, by which time overt signs of colitis were absent. This model induces colonic nociceptor hypersensitivity, enhanced dorsal horn neuronal activation, and allodynia and hyperalgesia to CRD.^41−44,77^ Therefore, these mice are termed as CVH mice.

### In Vivo VMR to CRD

Electromyography (EMG) recordings of abdominal contractions to CRD allow the assessment of visceral sensitivity in vivo in fully awake animals, as described in detail previously.^41−43,77^ Briefly, EMG recordings from electrodes sutured into the right abdominal muscle were performed in response to CRD applied at 20, 40, 50, 60, and 80 mmHg (each for a 20 s duration) with a barostat (Isobar 3, G&J Electronics) attached to a 2.5 cm balloon. The analogue EMG signal was rectified and integrated using Spike2 (Cambridge Electronic Design, UK). On the day of VMR assessment, healthy or CVH mice were briefly anesthetized using isoflurane and then administered a 100 μL enema of either a vehicle (sterile saline) or helianorphin-19 (10 μM). The first distension pressure commenced 10 min after administration, with the last distension pressure concluding 30 min after administration. VMR data (area under the curve, AUC) are presented as mean ± SEM, where N represents the number of animals. VMR data (AUC) were statistically analyzed by 2-way RM ANOVA with Sidak’s multiple-comparison tests using GraphPad Prism 9 software (San Diego, CA, USA). The cumulative AUC was quantified by adding together the individual AUC at each distension pressure for a total score per mouse.^43,44^ Cumulative AUC data are presented as mean ± SEM, where *N* represents the number of animals. Cumulative AUC data were analyzed with a Kruskal Wallis test and Dunn’s multiple-comparison test (GraphPad Prism 9). Statistical significance is reported as *P < 0.05 or ***P < 0.001.

### Ex Vivo Assessment of Colonic Nociceptor Mechanosensitivity

Single-unit extracellular recordings from splanchnic colonic afferent nerves were performed as previously described.^42,43,45,46,78^ CVH mice were humanely sacrificed, and the colon (5–6 cm) and mesentery (containing the lumbar colonic nerves) were removed intact with the attached neurovascular bundle containing the inferior mesenteric ganglion and lumbar splanchnic nerve.^46^ The tissue was transferred to ice-cold Krebs solution, and after further dissection, the distal colon was opened longitudinally and pinned flat with its mucosal side up in a specialized, SYLGARD 184-lined (Dow Corning Corp., Midland, MI) organ bath consisting of two adjacent, clear acrylic compartments (Danz Instrument Service, Adelaide, South Australia, Australia). The neurovascular bundle containing the splanchnic nerve was extended from the tissue compartment and laid onto a mirror in the recording compartment. A movable wall with a small “mouse hole” was lowered into position to allow passage of the nerves, and the recording chamber was filled with paraffin oil. The colonic compartment was superfused with a modified Krebs solution (117.9 mM NaCl, 4.7 mM KCl, 25 mM NaHCO_3_, 1.3 mM NaH_2_PO_4_, 1.2 mM MgSO_4_(H_2_O)7, 2.5 mM CaCl_2_, and 11.1 mM d-glucose) and gassed with carbogen (95% O_2_ and 5% CO_2_) at a temperature of 34 °C. Smooth muscle activity and endogenous prostaglandins were suppressed by the addition of the L-type calcium channel antagonist nifedipine (1 μM) and prostaglandin synthesis inhibitor indomethacin (3 μM), respectively. The splanchnic nerve was dissected away from the neurovascular bundle and the splanchnic nerve sheath using a dissecting microscope, and the nerve trunk was divided into three–eight bundles using fine forceps. Nerve bundles were individually placed onto a platinum recording electrode, while the platinum reference electrode rested on the mirror in a small pool of Krebs solution adjacent to the recording electrode. Receptive fields were identified by systematic stroking of the mucosal surface with a stiff brush, and mechanosensory response of the colonic nociceptor ending was examined using focal compression of the receptive field with calibrated von Frey filaments (2 g; each force applied three times for a period of 3 s with a 10 s interval between each application). After the baseline firing rate was recorded (response to mechanical stimulation with von Frey filaments [2 g]), helianorphin-19 (1 μM) and/or a selective KOR antagonist (0.1 μM nor-BNI alone and/or 0.1 μM nor-BNI plus 1 μM helianorphin-19) was applied for 5 min to the surface of the mucosal epithelium of identified colonic nociceptors. Measurement of the firing rate in response to mechanical stimulation with von Frey filaments (2 g) was repeated after drug application. Drugs were applied via a small metal ring placed on top of the receptor field, as previously described.^25,26,79^

### Rotarod Test

Helianorphin-19 and U50,488 were dissolved in saline and were administered with intraperitoneal (i.p.) injection at a dose of 5 mg/kg. The injection volume was 10 μL/g. Vehicle-treated mice were injected i.p. with 0.9% NaCl at the same injection volume. The rotarod test was performed as previously described.^80^ The latency to fall from the rotarod was automatically recorded (MedAssociates Inc., St. Albans, USA). Briefly, mice were first habituated to the rotating drum, and the speed of the drum was gradually increased from 4 to 40 rounds per min. Every mouse was subjected to the rotarod test three times, and the averaged latency to fall in the three trials was calculated. The intertrial interval was set to 15 min. The first trial began 45 min after the i.p. injection.

### Jump-Flinch Test

The jump-flinch test was performed according to a previously published protocol^81^ in a chamber capable of delivering electrical foot shocks of varying intensities (Med. Associates Inc. St. Albans, VT). The jump-flinch test was performed 60 min after the i.p. injections. Mice were initially given one min to explore the chamber, and then, they were presented with a single foot shock (duration of 1 s) of varying intensity every 30 s. Six different shock intensities were tested. Shocks began at an intensity of 0.1 mA and were gradually increased to a maximum intensity of 0.6 mA (0.1, 0.2, 0.3, 0.4, 0.5, and 0.6 mA). The flinch, the vocalizations, and the jump response (with all four paws) induced by the foot shocks were manually scored in a blinded manner. The minimum shock intensity needed to elicit the aforementioned responses was calculated and used to compare drug effects.^81^ Some of the animals failed to exhibit a jump response even at the highest intensity (0.6 mA), and in this case, the behavior was auto-scored with the highest possible intensity (0.6 mA).

## Supporting Information Available

Pharmacological data of helianorphins from functional cAMP and radioligand binding studies; stability data of helianorphin-19; HPLC and MALDI-MS chromatograms of helianorphin-19; one-dimensional ^1^H NMR spectra of helianorphins and ^13^C NMR spectrum of helianorphin-19; and HPLC purity, HPLC retention times, and observed mass (ESI- and MALDI-MS) of helianorphins (PDF)

## Abbreviations

GPCR: G protein-coupled receptor
KOR: κ-opioid receptor
MOR: μ-opioid receptor
DOR: δ-opioid receptor
SFTI: sunflower trypsin inhibitor
dyn A: dynorphin A
DPN: diprenorphine
HEK: human embryonic kidney
cAMP: cyclic adenosine monophosphate
BRET: bioluminescence resonance energy transfer
GRK: GPCR kinase
EGFP: enhanced green fluorescence protein
CVH: chronic visceral hypersensitivity
CRD: colorectal distention
VMR: visceromotor response
AUC: area under curve
RM ANOVA: repeated measures–analysis of variance
DNBS: dinitrobenzene sulfonic acid
EMG: electromyography
RP-HPLC: reversed-phase high-performance liquid chromatography
UPLC: ultraperformance liquid chromatography
ESI-MS: electrospray ionization mass spectrometry
MALDI-MS: matrix-assisted laser desorption ionization mass spectrometry
NMR: nuclear magnetic resonance
DMEM: Dulbecco’s modified Eagle medium
LC–MS: liquid chromatography–mass spectrometry
SGF: simulated gastric fluid
