# Supplementary material for: Design of a Stable Cyclic Peptide Analgesic Derived from Sunflower Seeds that Targets the κ-Opioid Receptor for the Treatment of Chronic Abdominal Pain
Source: J Med Chem. 2021 Jun 24;64(13):9042–55. doi: 10.1021/acs.jmedchem.1c00158 (PMC8273886; doi:10.1021/acs.jmedchem.1c00158)
Supplement: Supplementary file 1 — jm1c00158_si_001.pdf [file jm1c00158_si_001.pdf]

Supporting Information

Design of a stable cyclic peptide analgesic derived from sunflower seeds that targets the κ-opioid receptor for the treatment of chronic abdominal pain

Edin Muratspahić, Nataša Tomašević, Johannes Koehbach, Leopold Duerrauer, Seid Hadžić, Joel Castro, Gudrun Schober, Spyridon Sideromenos, Richard J. Clark, Stuart M. Brierley, David J. Craik, Christian W. Gruber*

***Email:** christian.w.gruber@meduniwien.ac.at

This PDF file includes:

Figures S1 to S22
Table S1

Table of contents

Figure S1………. S2
Figure S2.. .... S3
Figure S3 .. .. S4
Figure S4.. .... S5
Figure S5.. .... S6
Figure S6.. .... S7
Figure S7 .... S8
Figure S8.. .... S9
Figure S9.. .... S10
Figure S10.. .... S11
Figure S11.. .... S12
Figure S12.. .... S13
Figure S13.. .... S14
Figure S14.. .... S15
Figure S15.. .... S16
Figure S16.. .... S17
Figure S17.. .... S18
Figure S18.. .... S19
Figure S19.. .... S20
Figure S20.. .... S21
Figure S21.. .... S22
Figure S22.. .... S23
Table S1.. .... S24

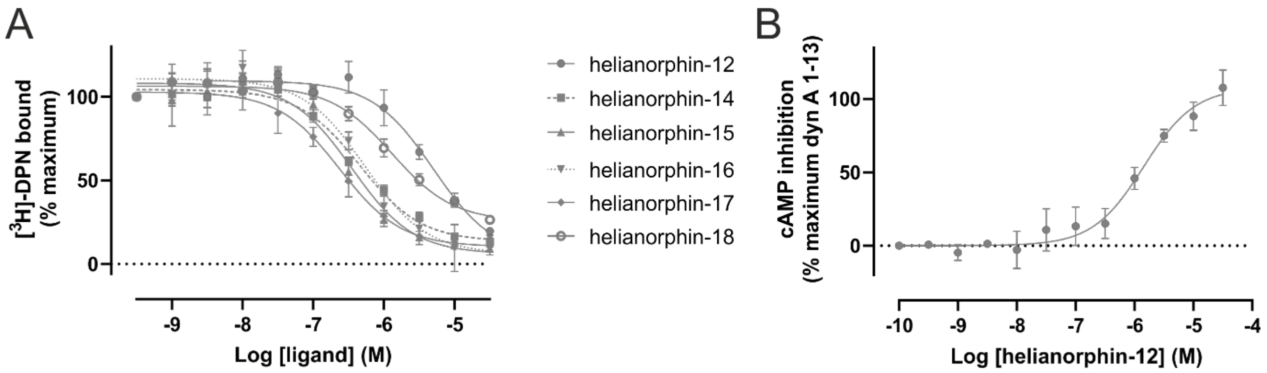

Figure S1. *(A)* Concentration-dependent displacement of [^{3}H]-diprenorphine (1 nM; DPN) by helianorphin-12 and 14-18 in HEK293 cells stably expressing mouse KOR (n=3). To determine specific binding, nonspecific binding was subtracted from total binding. *(B)* cAMP inhibition was measured following mouse KOR activation by helianorphin-12 (n=4). Data are normalized to 100% and are mean ± SD.

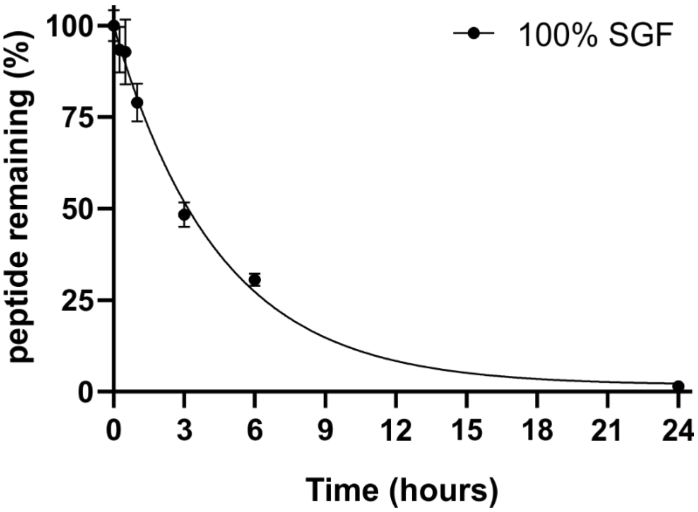

Figure S2. Stability of helianorphin-19 was determined by UPLC-MS after incubation in 100% simulated gastric fluid (SGF) (n=3).

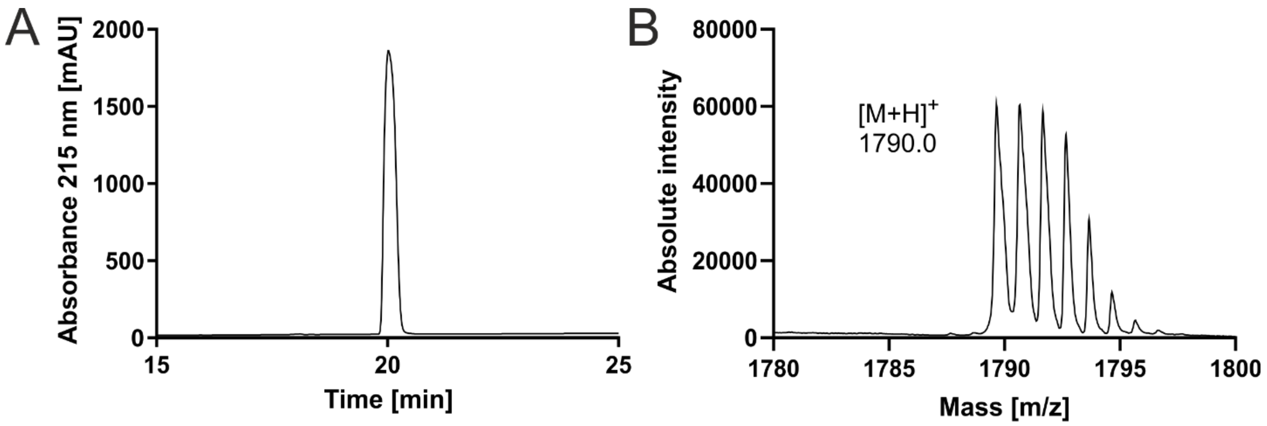

Figure S3. Quality control of helianorphin-19 was determined by *(A)* RP-HPLC and *(B)* MALDI mass spectrometry. The chromatogram at 215 nm indicates a purity >95% and the monoisotopic mass signal (1790.0 m/z) represents oxidized cyclic peptide.

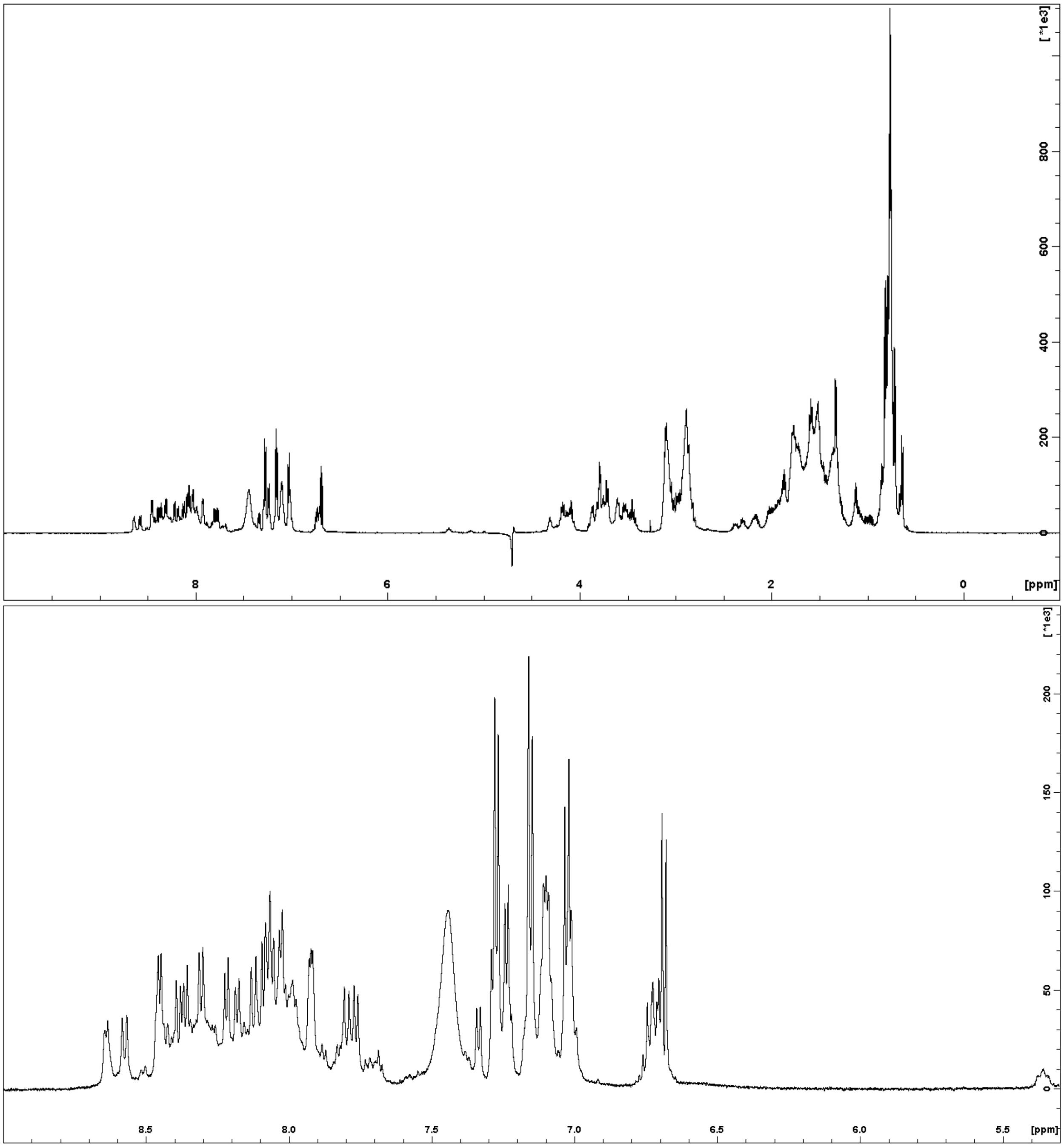

Figure S4. Full one-dimensional ^{1}H NMR spectrum (0-10 ppm) of helianorphin-2.

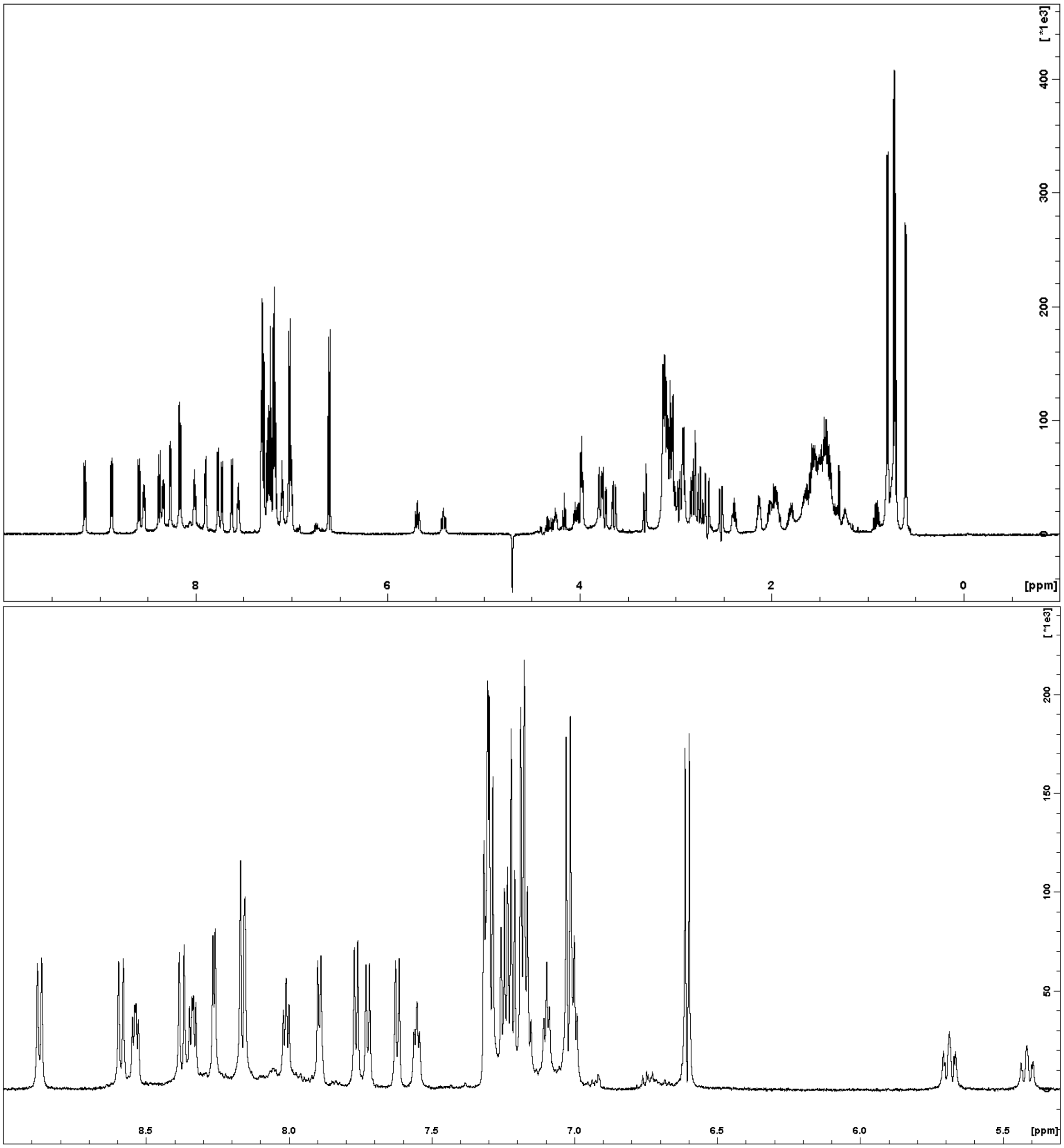

Figure S5. Full one-dimensional ^{1}H NMR spectrum (0-10 ppm) of helianorphin-3.

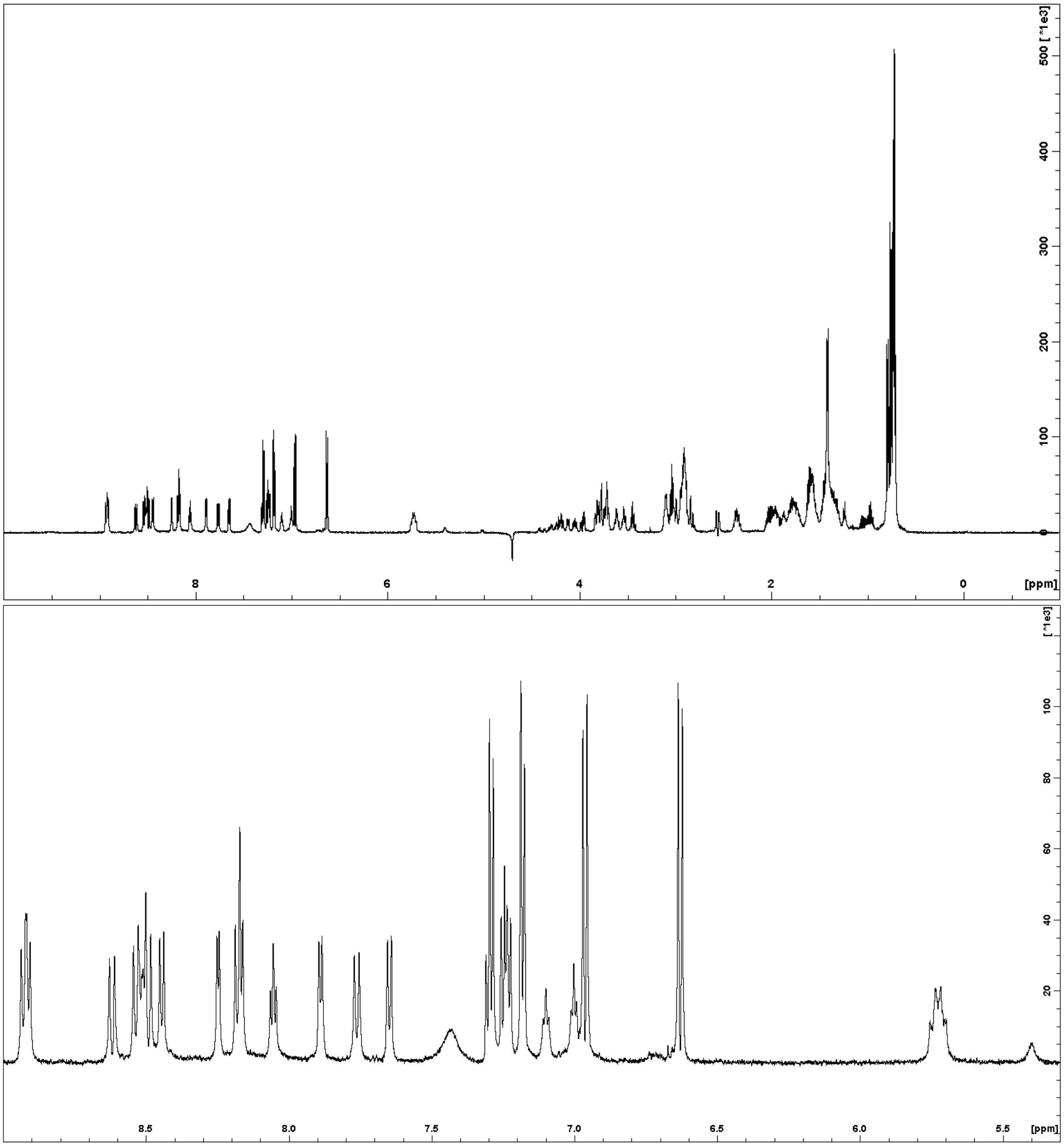

Figure S6. Full one-dimensional ^{1}H NMR spectrum (0-10 ppm) of helianorphin-4.

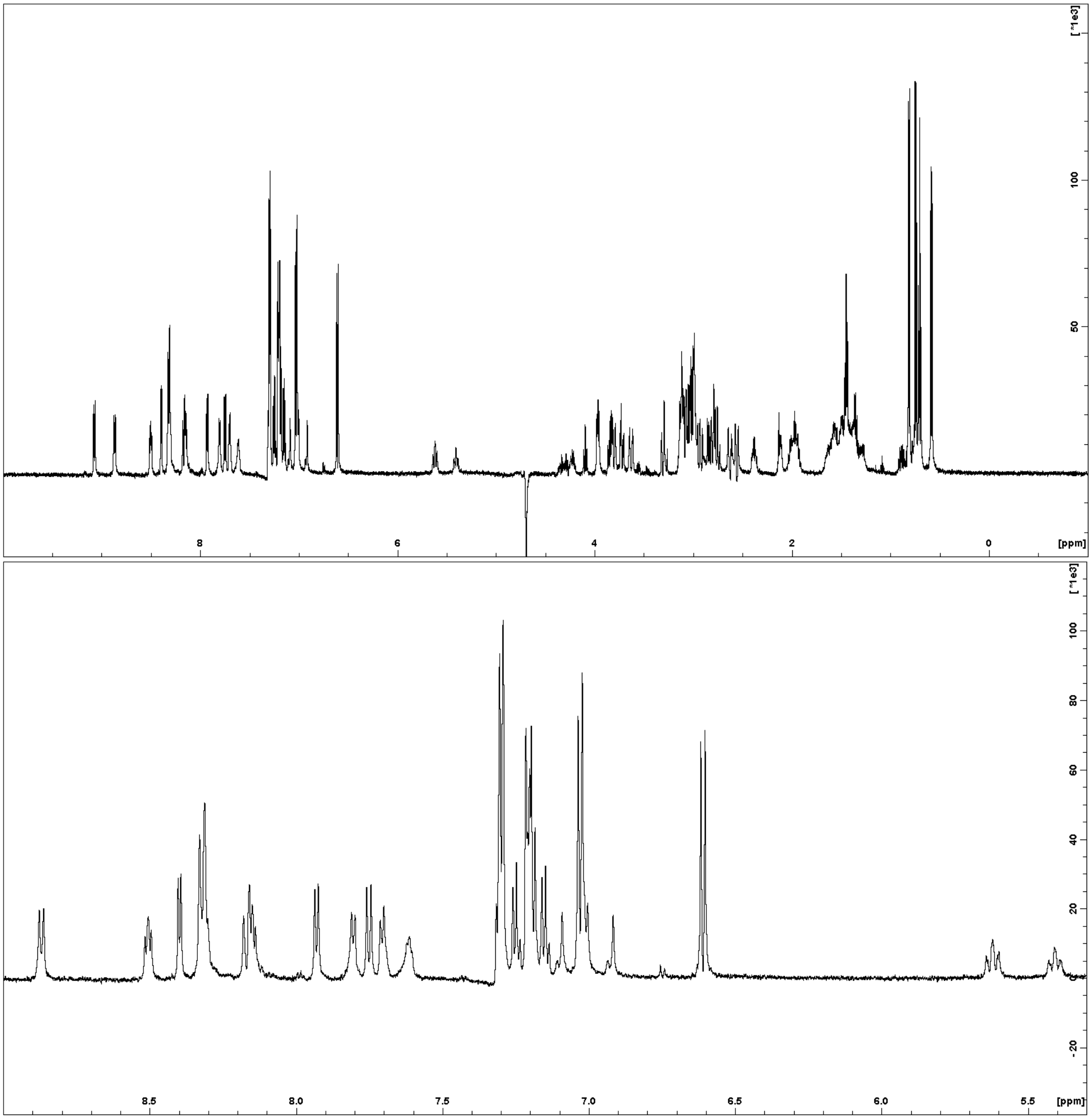

Figure S7. Full one-dimensional ^{1}H NMR spectrum (0-10 ppm) of helianorphin-5.

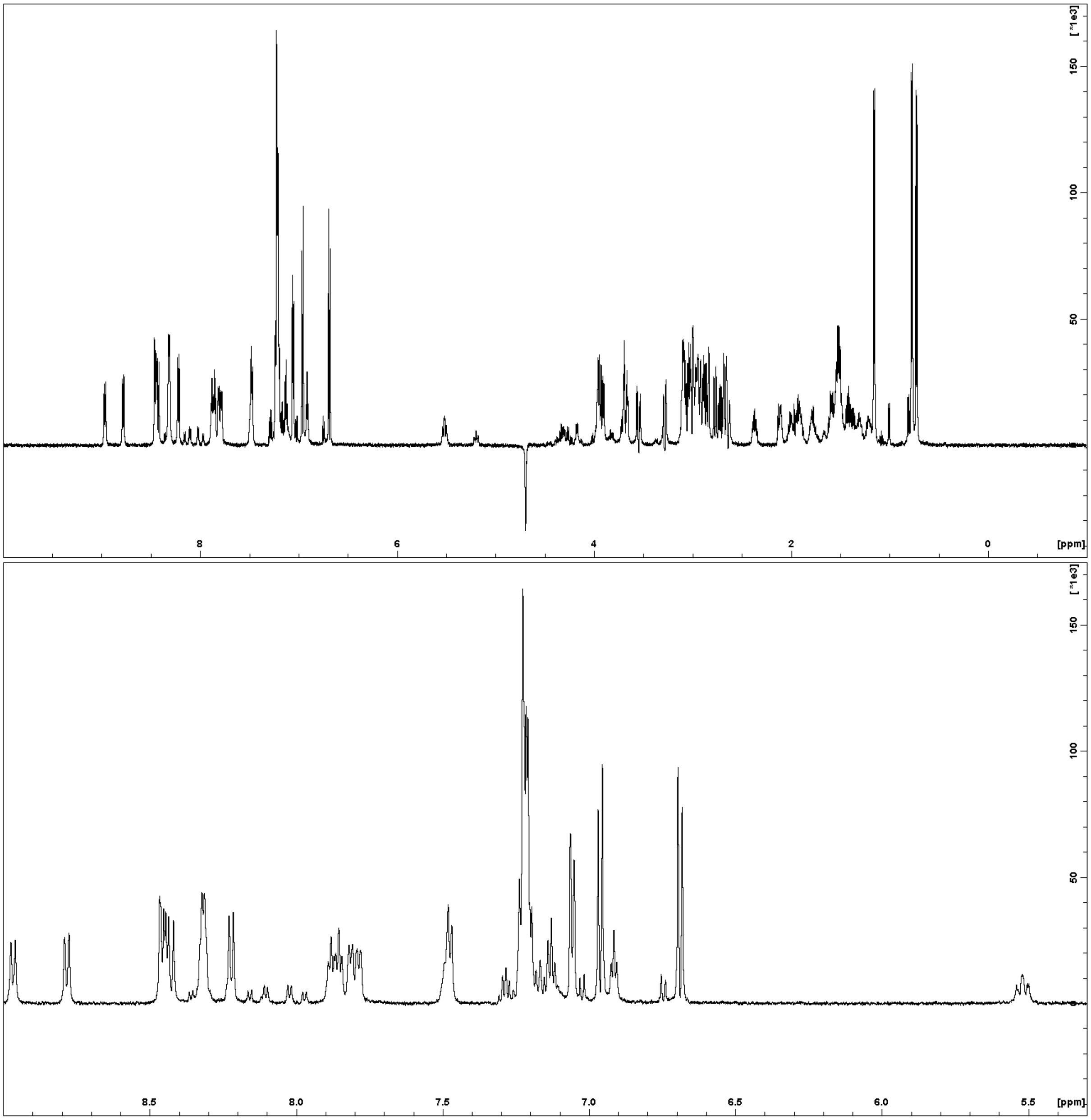

Figure S8. Full one-dimensional ^{1}H NMR spectrum (0-10 ppm) of helianorphin-6.

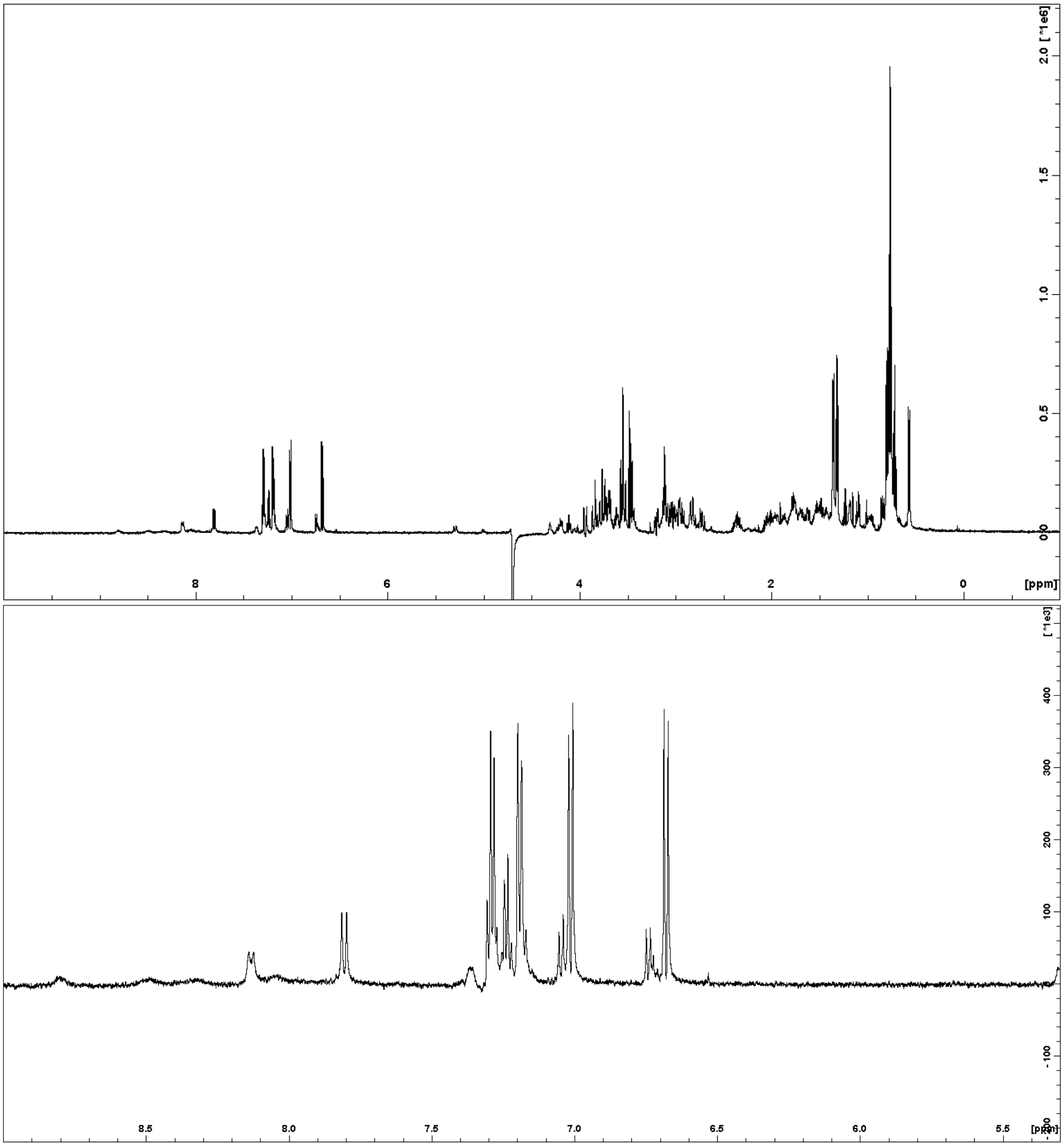

Figure S9. Full one-dimensional ^{1}H NMR spectrum (0-10 ppm) of helianorphin-7.

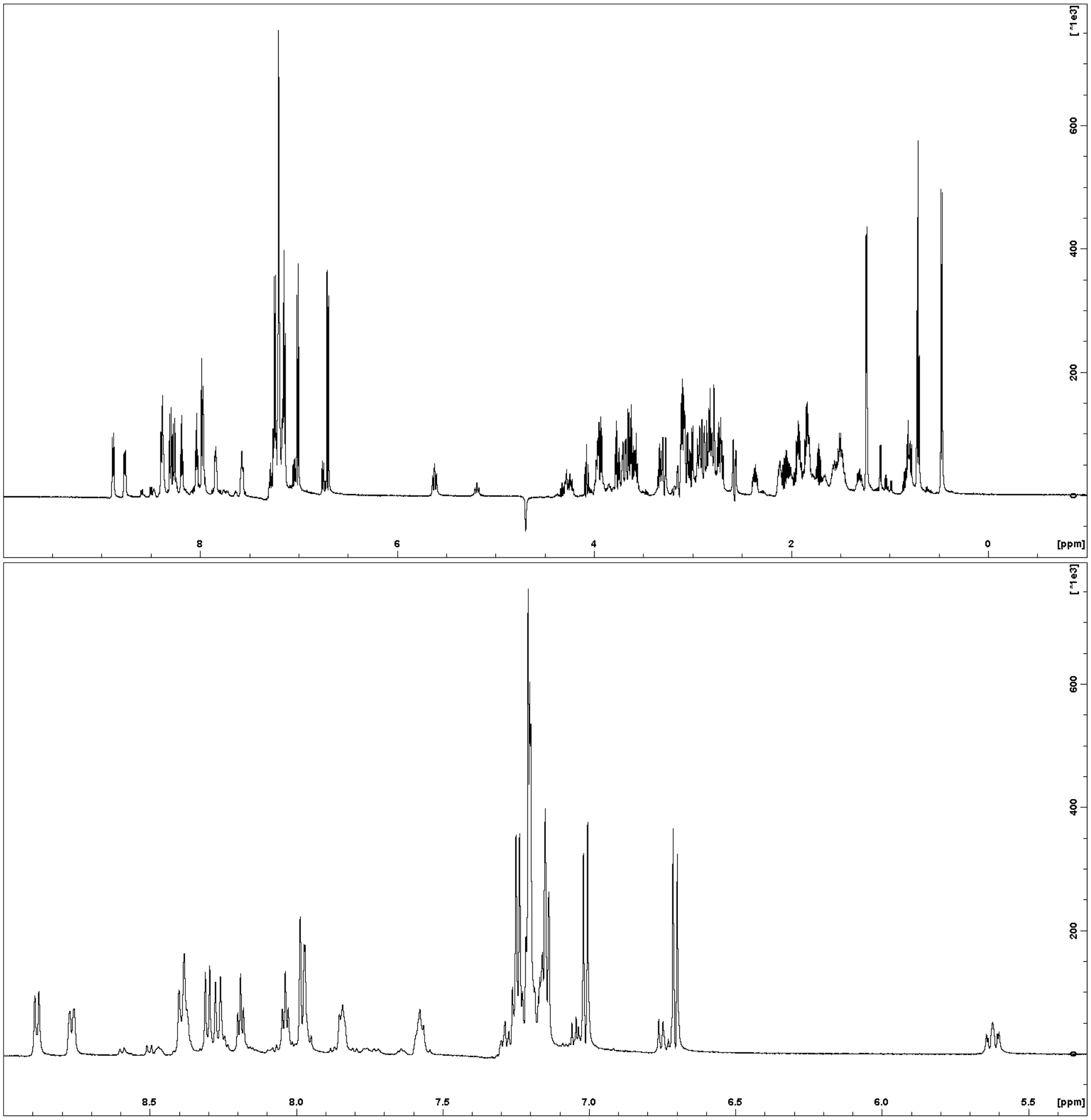

Figure S10. Full one-dimensional ^1H NMR spectrum (0-10 ppm) of helianorphin-8.

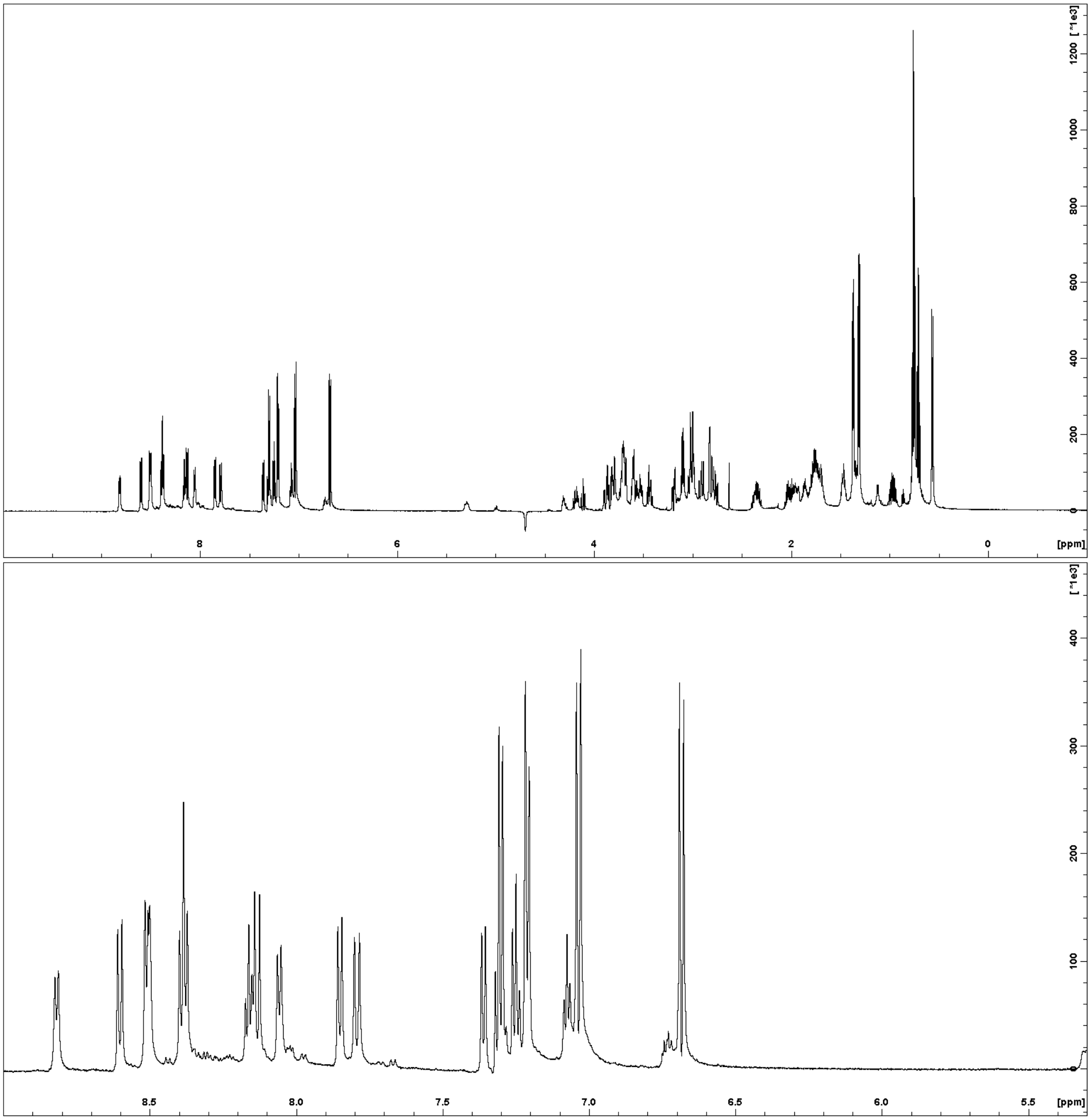

Figure S11. Full one-dimensional ^{1}H NMR spectrum (0-10 ppm) of helianorphin-9.

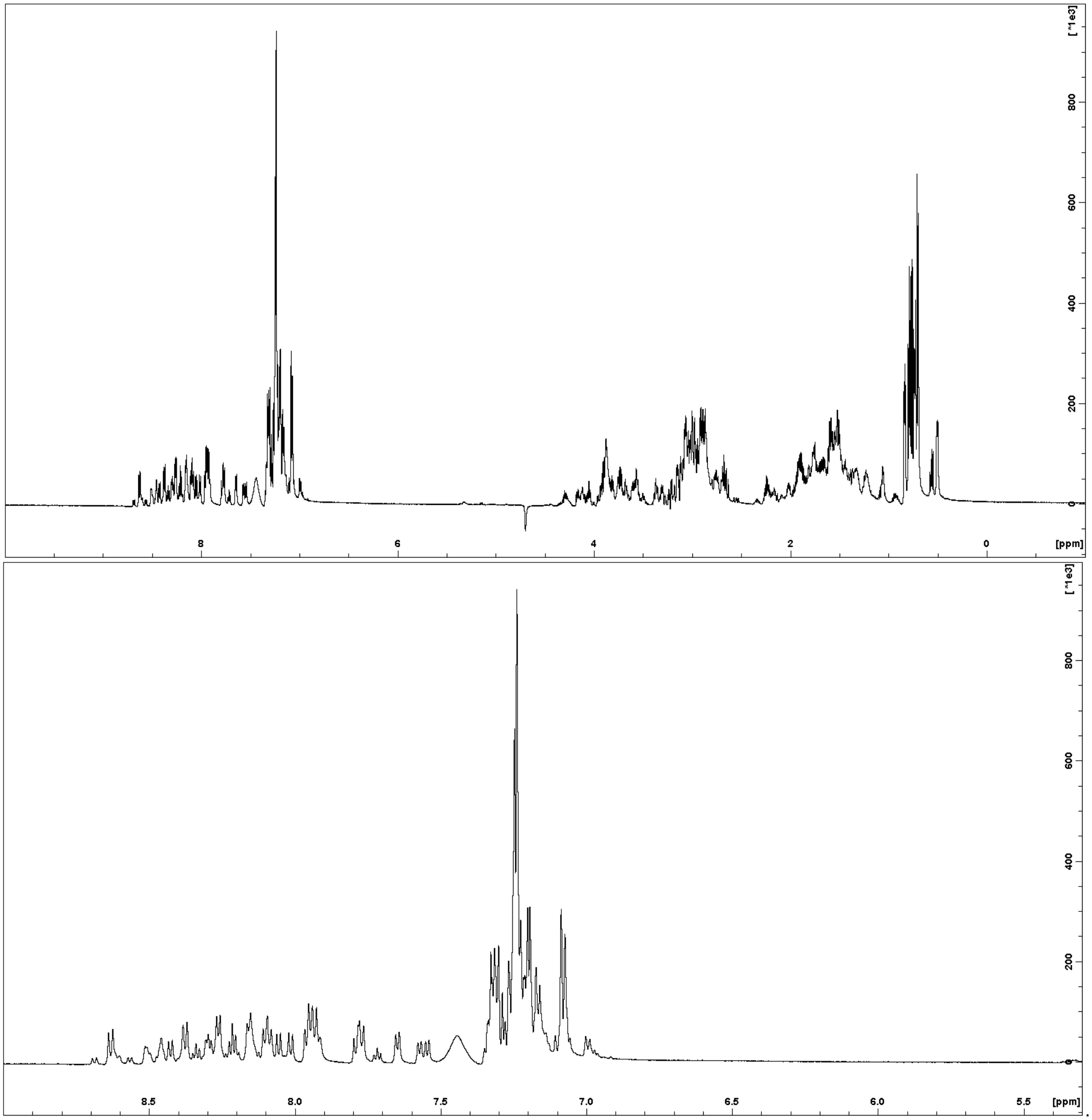

Figure S12. Full one-dimensional ^{1}H NMR spectrum (0-10 ppm) of helianorphin-10.

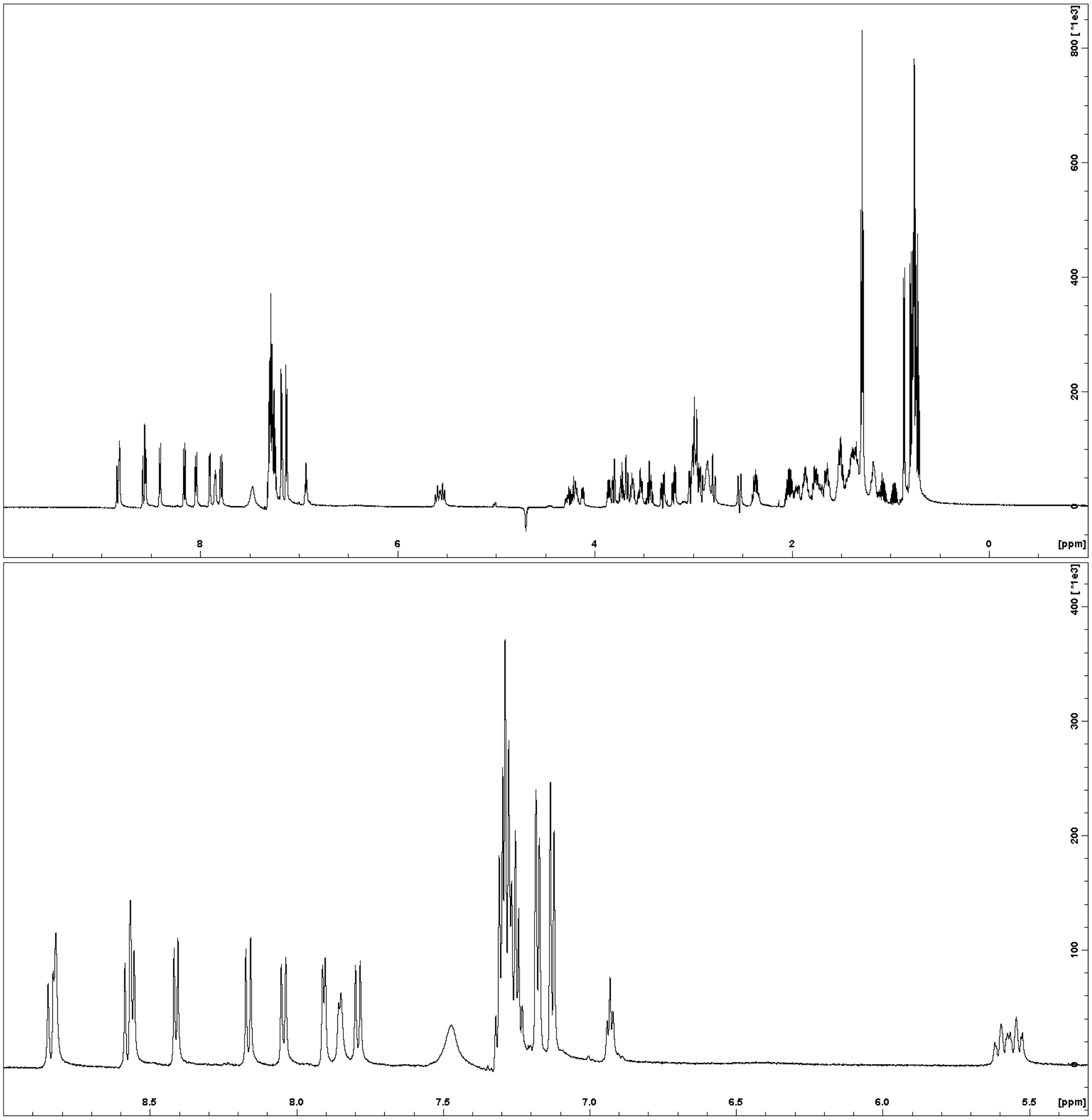

Figure S13. Full one-dimensional ^{1}H NMR spectrum (0-10 ppm) of helianorphin-11.

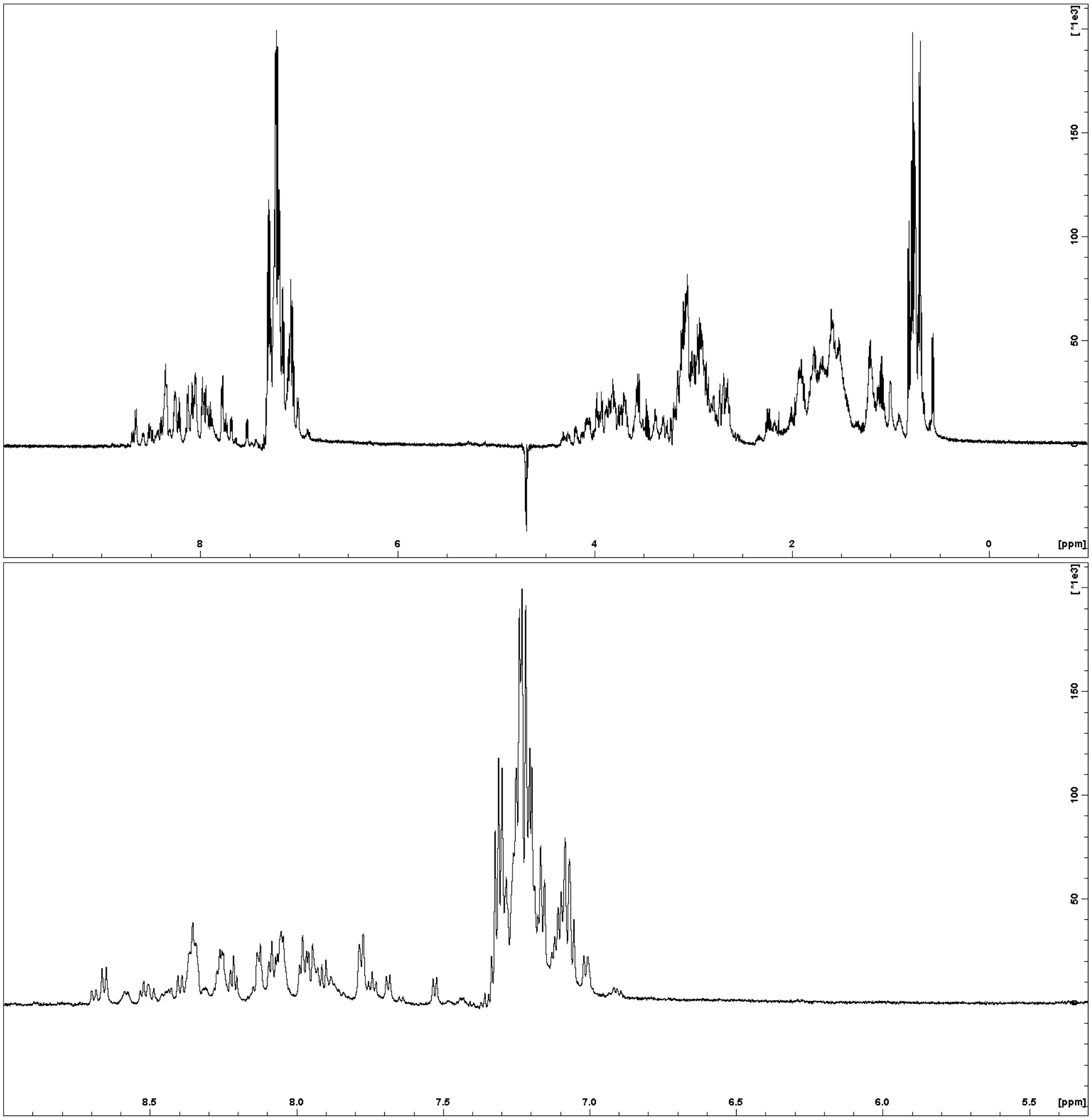

Figure S14. Full one-dimensional ^{1}H NMR spectrum (0-10 ppm) of helianorphin-12.

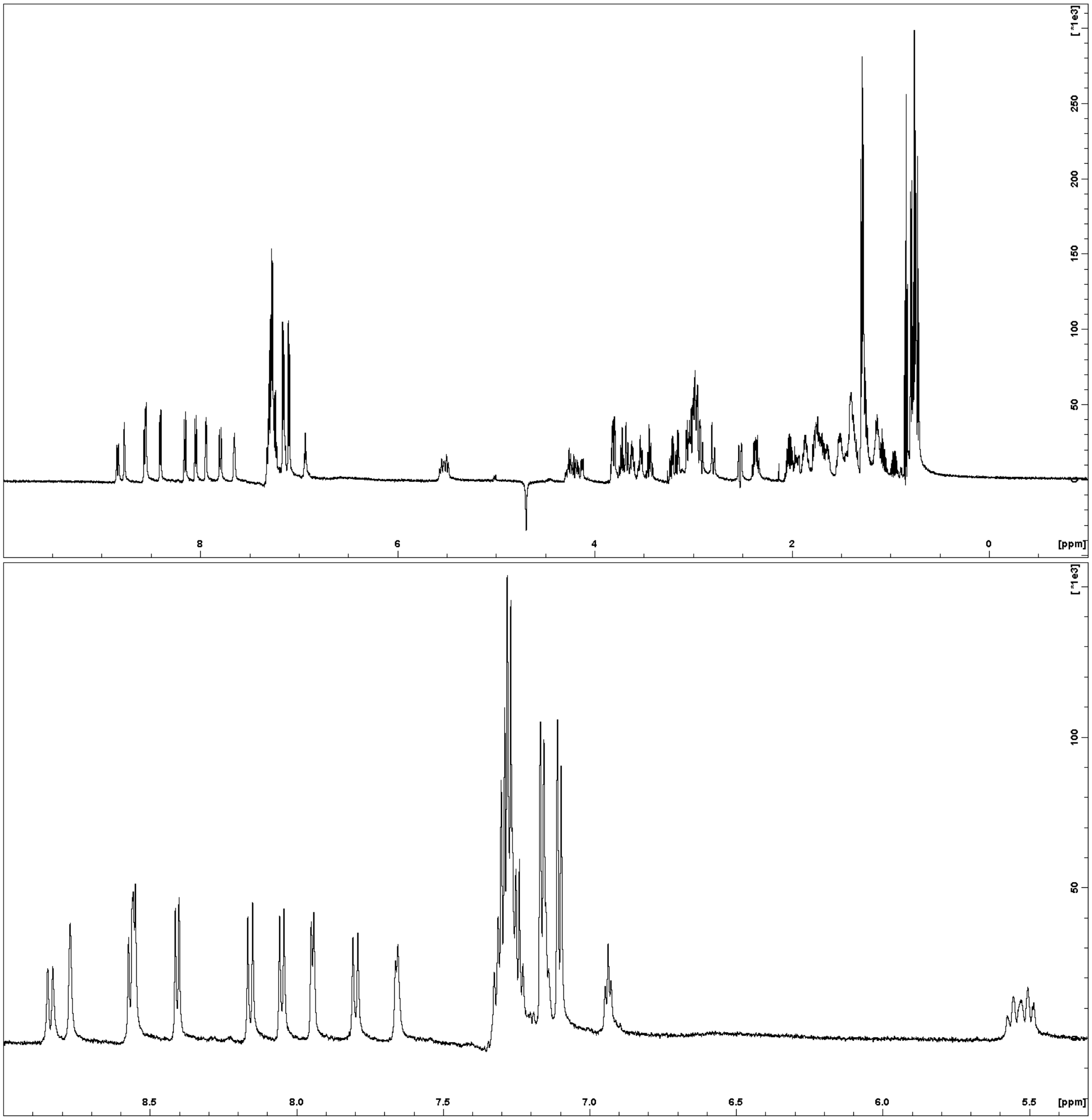

Figure S15. Full one-dimensional ^{1}H NMR spectrum (0-10 ppm) of helianorphin-13.

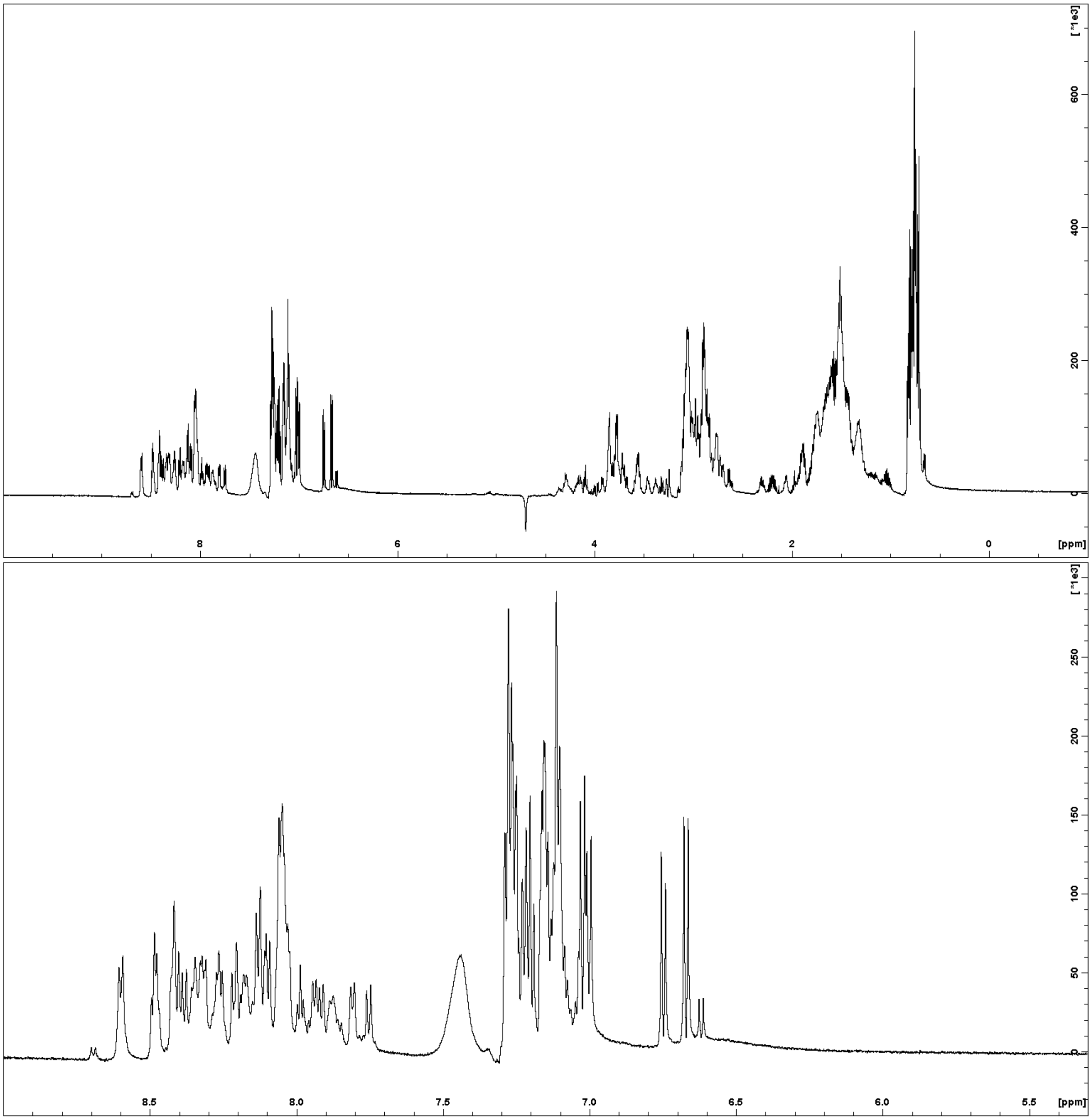

Figure S16. Full one-dimensional ^{1}H NMR spectrum (0-10 ppm) of helianorphin-14.

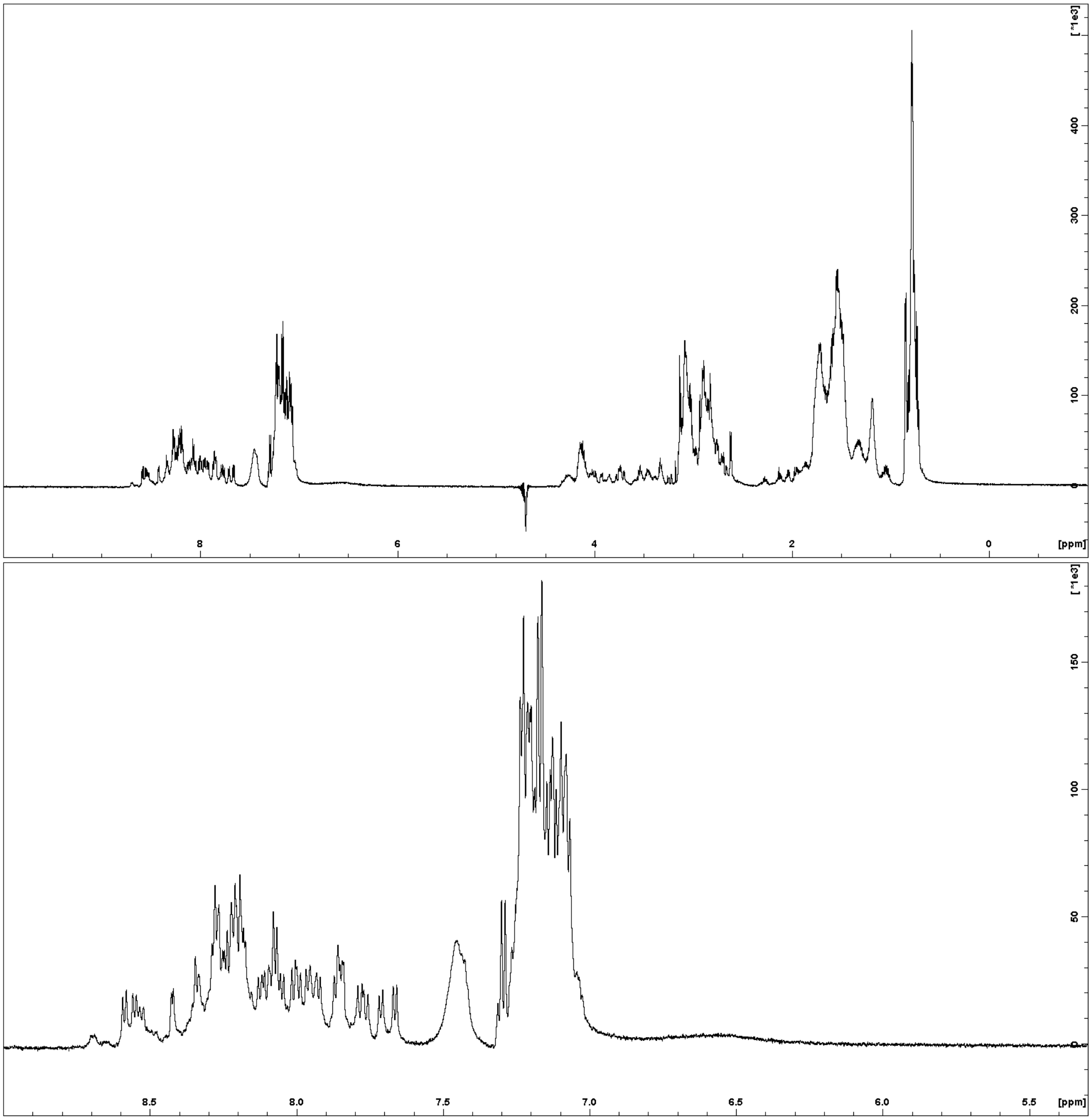

Figure S17. Full one-dimensional ^{1}H NMR spectrum (0-10 ppm) of helianorphin-15.

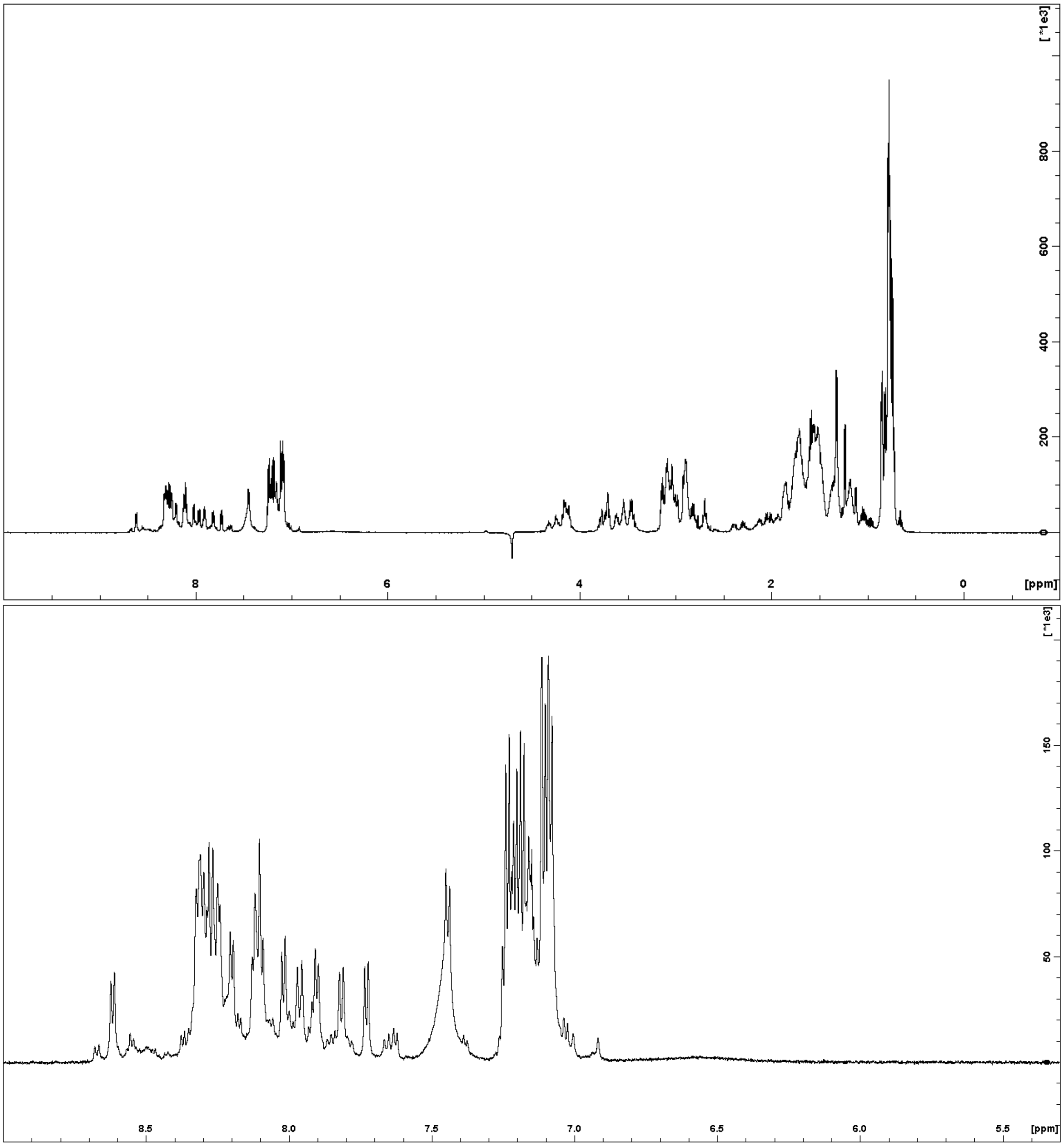

Figure S18. Full one-dimensional ^{1}H NMR spectrum (0-10 ppm) of helianorphin-16.

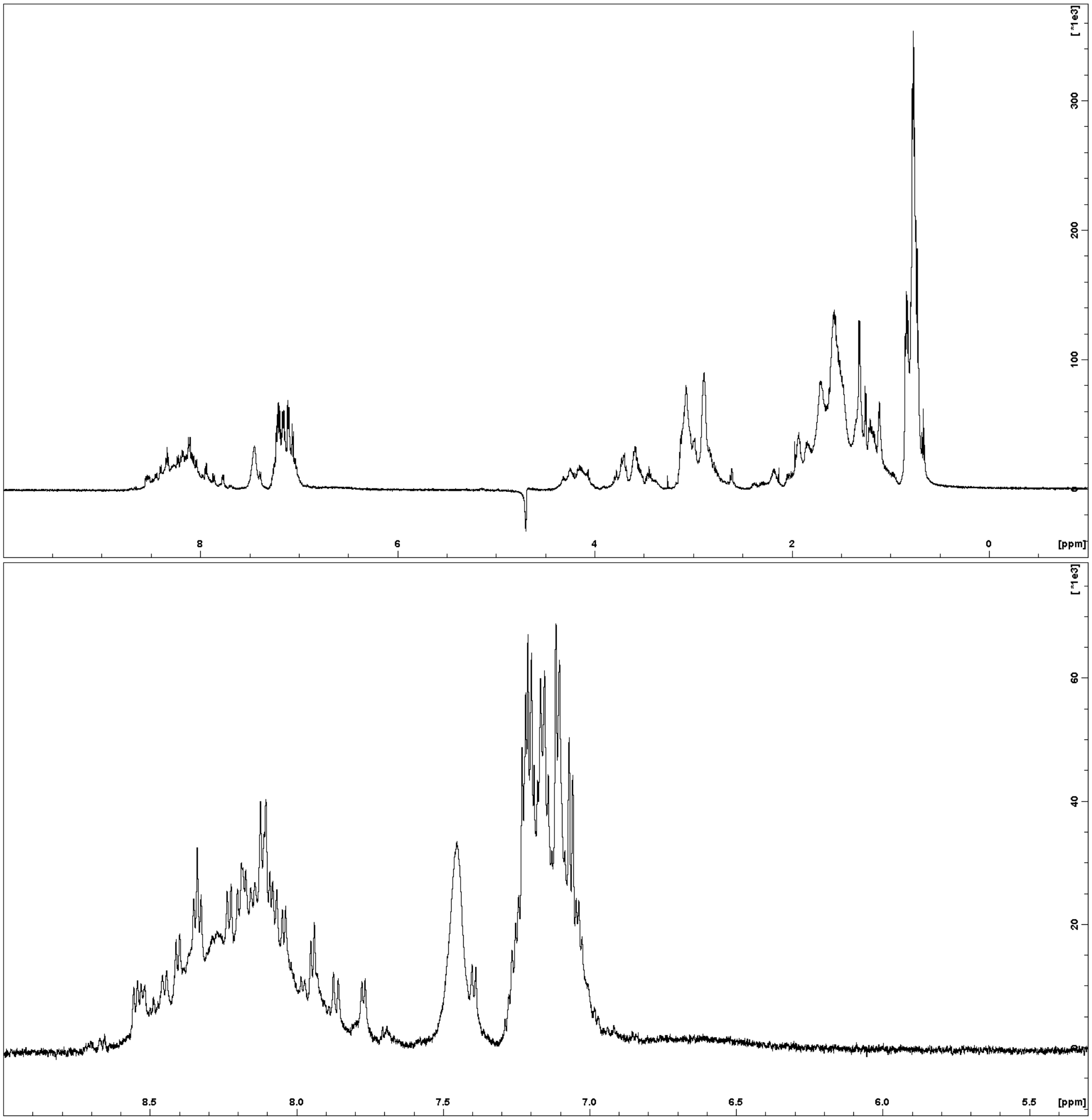

Figure S19. Full one-dimensional ^1H NMR spectrum (0-10 ppm) of helianorphin-17.

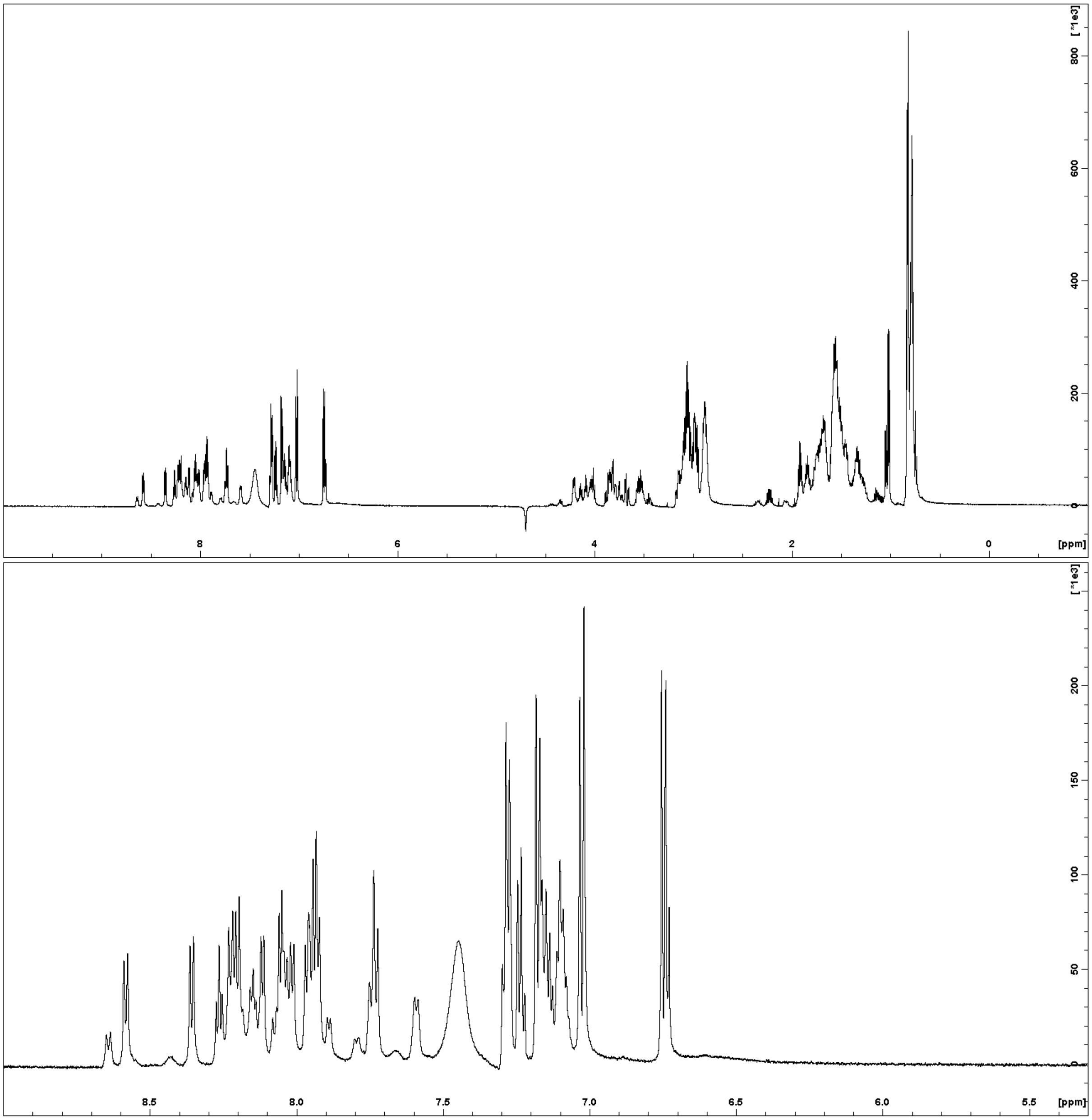

Figure S20. Full one-dimensional ^{1}H NMR spectrum (0-10 ppm) of helianorphin-18.

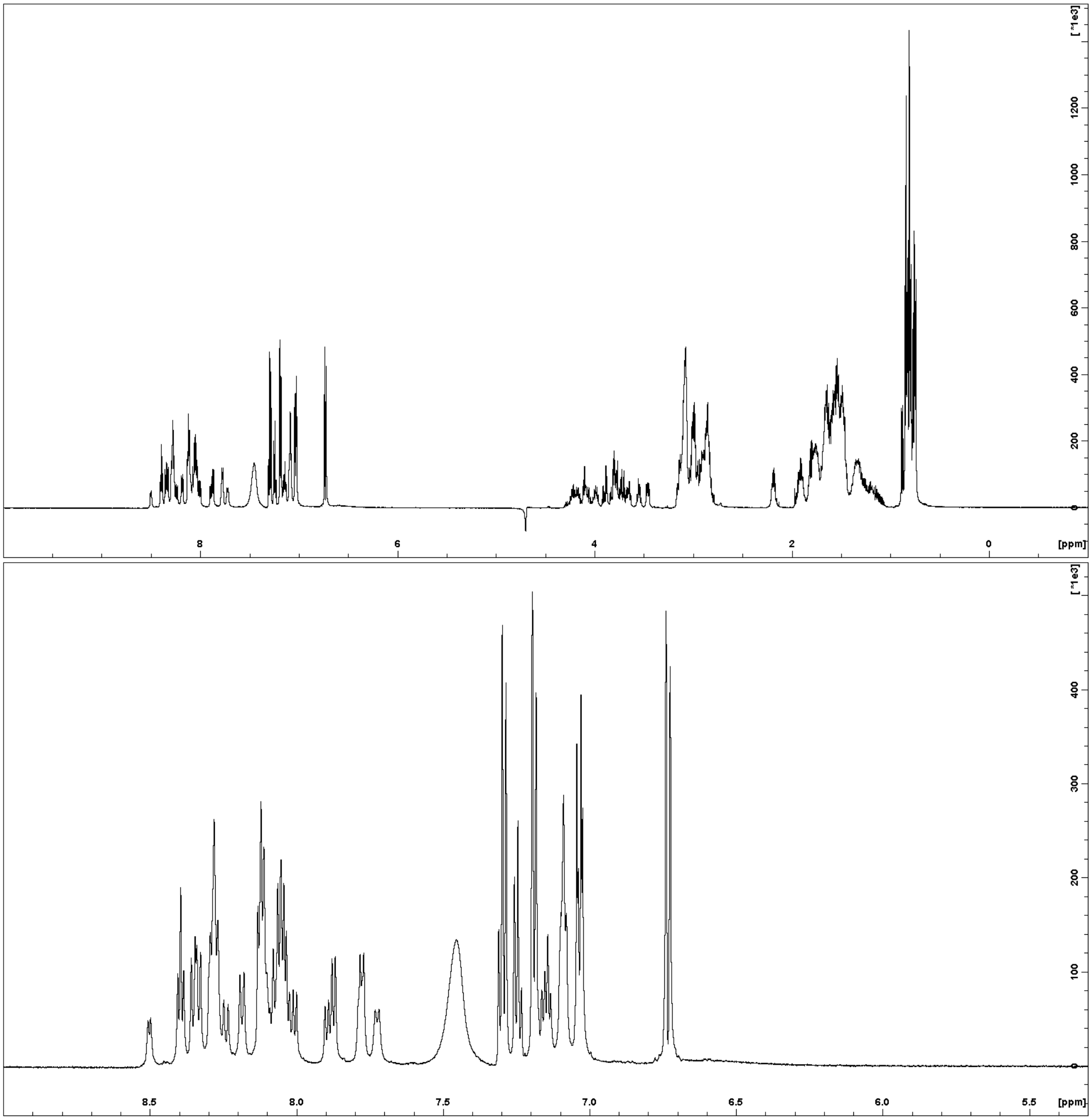

Figure S21. Full one-dimensional ^{1}H NMR spectrum (0-10 ppm) of helianorphin-19.

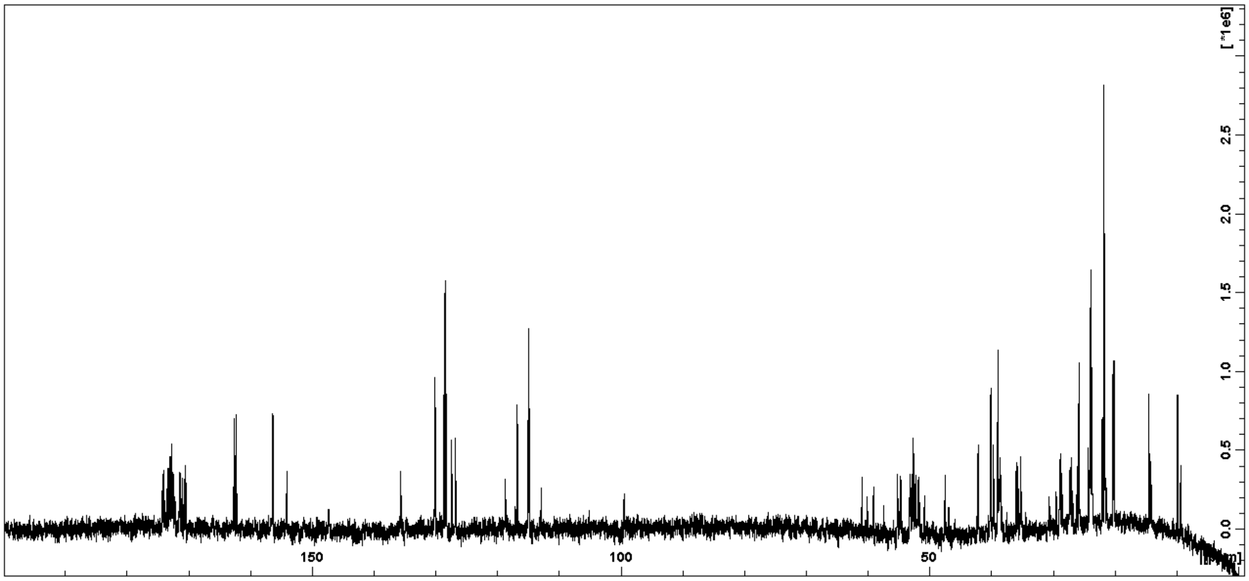

Figure S22. Full one-dimensional ^{13}C NMR spectrum (0-200 ppm) of helianorphin-19.

Table S1. Analytical data of synthesized peptides.

Peptide	HPLC purity (%)*	HPLC retention times (min)	Calculated mass (m/z)	Observed mass (m/z)#
SFTI-1	100.0	36.9	1513.8	1513.6
helianorphin-1	99.8	33.4	2362.3	2362.0
helianorphin-2	99.7	37.0	2526.5	2526.1
helianorphin-3	99.9	38.5	1739.9	1739.6
helianorphin-4	96.0	37.6	1904.0	1903.8
helianorphin-5	99.3	10.5	1583.8	1583.8
helianorphin-6	99.3	7.6	1571.7	1571.7
helianorphin-7	100.0	10.1	1512.7	1512.7
helianorphin-8	99.5	9.2	1623.8	1623.8
helianorphin-9	98.3	8.8	1651.8	1651.9
helianorphin-10	94.2	10.0	1577.8	1577.8
helianorphin-11	93.5	10.2	1464.7	1464.7
helianorphin-12	100.0	9.8	1575.8	1575.8
helianorphin-13	100.0	10.5	1603.8	1603.8
helianorphin-14	98.9	7.2	2362.3	2362.2
helianorphin-15	99.5	7.8	2501.4	2501.4
helianorphin-16	100.0	9.6	2608.5	2608.5
helianorphin-17	100.0	9.4	2608.5	2608.9
helianorphin-18	99.1	7.3	1891.1	1891.4
helianorphin-19	99.9	20.0	1790.0	1790.0

*Purity of SFTI-1 and helianorphin 1-4 and 19 (calculated by automatic peak integration from 5-45 min) was determined by RP-HPLC using a Phenomenex Jupiter C_{18} column (5 µM, 300 Å, 150 x 2 mm) and a linear gradient of 5-65% solvent B in 60 min at a flow rate of 1 mL/min. Purity of helianorphin 5-18 was assessed by RP-UPLC using a Phenomenex Luna Omega column (1.6 µm C_{18} 100 Å, 50 x 2.1 mm) and a linear gradient of 1-61% of solvent B in 15 min at a flowrate of 0.6 mL/min was applied. Purity was calculated by automatic peak integration from 3-15 min.

#Monoisotopic masses of peptides obtained by ESI- and/or MALDI-MS are shown.
